# Assessing the current and potential future distribution of four invasive forest plants in Minnesota, U.S.A., using mixed sources of data

**DOI:** 10.1038/s41598-020-69539-1

**Published:** 2020-07-29

**Authors:** Jason R. Reinhardt, Matthew B. Russell, Senait Senay, William Lazarus

**Affiliations:** 1 Department of Forest Resources, University of Minnesota, Minneapolis, MN USA; 2 Department of Plant Pathology, University of Minnesota, Minneapolis, MN USA; 3 Department of Applied Economics, University of Minnesota, Minneapolis, MN USA

**Keywords:** Forest ecology, Invasive species

## Abstract

Invasive plants are an ongoing subject of interest in North American forests, owing to their impacts on forest structure and regeneration, biodiversity, and ecosystem services. An important component of studying and managing forest invaders involves knowing where the species are, or could be, geographically located. Temporal and environmental context, in conjunction with spatially-explicit species occurrence information, can be used to address this need. Here, we predict the potential current and future distributions of four forest plant invaders in Minnesota: common buckthorn (*Rhamnus cathartica*), glossy buckthorn (*Frangula alnus*), garlic mustard (*Alliaria petiolata*), and multiflora rose (*Rosa multiflora*). We assessed the impact of two different climate change scenarios (IPCC RCP 6.0 and 8.5) at two future timepoints (2050s and 2070s) as well as the importance of occurrence data sources on the potential distribution of each species. Our results suggest that climate change scenarios considered here could result in a potential loss of suitable habitat in Minnesota for both buckthorn species and a potential gain for *R. multiflora* and *A. petiolata*. Differences in predictions as a result of input occurrence data source were most pronounced in future climate projections.

## Introduction

Invasive plants have been a topic of concern in forest ecosystems for several decades, owing to their impacts on biodiversity, community composition, forest structure, and ecosystem services^1,2^. These impacts come as a result of altered regeneration and recruitment patterns, fire regimes, hydrology, and trophic interactions^2^. In the U.S., forests are affected by a number of different invaders, ranging from herbaceous plants like Japanese stiltgrass (*Microstegium vimineum*) to woody plants such as Japanese barberry (*Berberis thunbergii*), privet (*Ligustrum* spp.), tree-of-heaven (*Ailanthus altissima*), and honeysuckle (*Lonicera* spp.). These invaders have varying effects on U.S. forests. Japanese stiltgrass, for example, can form dense patches of homogenous growth under a wide range of light and moisture conditions, ultimately interfering with forest regeneration^3^. Bush honeysuckles (*Lonicera* spp.) are similar in that they can frequently form patches of dense homogenous growth, crowd out native vegetation^4,5^, and even alter the behavior of native wildlife^6,7^. Species-specific impacts on forest ecosystems vary^8,9^, and the species of concern can vary regionally. In the eastern and central U.S. in particular, common and glossy buckthorn (*Rhamnus cathartica*, *Frangula alnus*), garlic mustard (*Alliaria petiolata*), and multiflora rose (*Rosa multiflora*) are plants of concern and are the focus of this study.

*Rhamnus cathartica* and *F. alnus* are two morphologically similar woody invaders increasingly common in forests across the upper Midwest of the U.S. The more widespread of these, *R. cathartica*, invades a variety of habitat types including forests and forest edges^10,11^ and can tolerate drier upland conditions as well as some partially flooded conditions^12^. *R. cathartica* may affect native forests in several ways^11^, including reducing overall forest biomass relative to non-invaded stands^13^, reducing native understory plant germination, survival, and growth^14,15^ and altering leaf litter cover and forest floor communities^16,17^. *F. alnus* occupies a niche similar to *R. cathartica* but has been reported to invade more mesic and wet sites rather than upland sites^10,18^. Research examining the impacts of *F. alnus* on native forests suggests a variety of impacts, including reduced growth and survival of native plants, particularly tree seedlings^19^, reduced native tree seedling density^20,21^ and plant species abundance^20^. Taken together, these two woody invaders represent a challenge for conservation professionals, foresters, and land managers seeking to maintain healthy forests across the upper Midwest.

*Alliaria petiolata* and *R. multiflora* are forest understory invaders widespread throughout the eastern and central U.S.^22^. *Alliaria petiolata* is rare among nonnative herbaceous invasive plants in that it is able to successfully invade intact and shaded forest understories^23^, giving it the ability to affect forests beyond edges and gaps. It competes with native understory vegetation^24–26^ and some data suggests it may alter tree seedling abundance and composition^27^. Similar to *A. petiolata*, *R. multiflora* is able to invade intact understories in addition to its prevalence along forest edges and in gaps^28,29^. *Rosa multiflora* is a woody shrub which can form dense thickets in areas where it becomes established, inhibiting the growth of native species and making traversal by wildlife or humans difficult^30^. These two species have the capacity to alter forest ecosystems through impacts on regeneration abundance and composition, not dissimilar from some of the impacts of buckthorn. Indeed, all four species described here have elicited concern from conservation groups, state agencies, and land managers in multiple states across the upper Midwest.

The response to these and other invasive plants typically follows an invasion-dependent set of management strategies: (1) prevention, (2) early detection and eradication, and (3) mitigating the impacts of established invasives^31^. The management responses undertaken depend on the specifics of the invasion and forest types in question. In Minnesota, the area of interest for this study, all four of the species described above are present and have established populations, though not throughout the entire state^22^. Prevention efforts often focus on raising awareness about the invasive plants and in preventing the spread of seed, though for species such as buckthorn where fruits are frequently dispersed by birds^11,32^ preventing dispersal can be difficult. Government regulations are one of the primary tools used in prevention efforts. Early detection and eradication efforts typically focus on finding and destroying the invasive plants as they spread into new areas along the invasion front^33–35^, with a major challenge being detection over large or remote areas. Mitigation efforts for these four species in Minnesota may come in the form of forest restoration and/or forest management treatments including site-preparation, mechanical treatments and prescribed burning.

The invasive plant management strategies outlined here frequently depend on data-driven tools such as treatment prescription databases (e.g., Midwest Invasive Plant Network’s invasive plant control database^36^) frequently-updated reporting maps based on observations from the public and private community^22^, databases of systematically-collected invasive plant data, forest and land cover datasets, and species distribution models (SDMs)^37,38^. SDMs can be particularly useful because they can fill in the spatial gaps between reported plant sightings and illustrate potential areas of threat^37–39^, providing information that can aid the prevention and early detection management strategies. Such models can be sensitive to input data, however^38,40,41^, and this can become a concern when species location data are collected in an unsystematic way and are reported by different sources or pulled from different databases. In addition, SDMs are typically trained based on contemporary environmental conditions whereas invasive plant management is an ongoing and decades-long process. It is therefore important from a long-term planning perspective to consider not only the contemporary distributions as predicted by SDMs, but also future projections which take varying climate change scenarios into account.

In this study, we aimed to estimate the distribution of the four forest-invaders described above: *R. cathartica*, *F. alnus*, *A. petiolata*, and *R. multiflora* across the state of Minnesota. All four species are invasive in Minnesota forests, and spur ongoing management and control efforts in the state. We sought to compare current distribution estimates with future estimates under two different climate change emissions scenarios as defined by the Intergovernmental Panel on Climate Change (RCP 6.0 and 8.5)^42^, and to determine whether input data source and composition (i.e., observations from public, private and unattributed sources had an impact on current and future predictions as well as model accuracy. Specifically, our objectives were to: (1) model and estimate the current potential distribution of all four species across Minnesota, (2) analyze the potential impacts of climate change on the potential distribution of each species, and (3) determine the relative importance and contribution of plant reporting source to current and future estimates of potential distribution for each species.

## Methods

### Study area

This study was focused on invasive plant distributions and changes in distribution across the U.S. state of Minnesota. Ecosystems and land cover types vary across the state, ranging from boreal forests in the northeast, mixed hardwood and agricultural land in a region running from the north-central part of the state to the southeast corner, to ubiquitous cropland and heavily fragmented prairie in the west and south-west regions of the state. Forests in the north and northeast regions of the state consist of species such as pine (*Pinus* spp.), quaking aspen (*Populus tremuloides*), spruce (*Picea* spp.), balsam fir (*Abies balsamea*), and paper birch (*Betula papyrifera*). In the central and southeastern part of the state, species such as oak (*Quercus* spp.), maple (*Acer* spp.), quaking aspen, and basswood (*Tilia americana*) are common^43^. In the agricultural regions of the state, row cropland producing maize (> 3 million ha), soybeans (> 3 million ha), hay (~ 0.5 million ha), and wheat (> 0.6 million ha) are most common^44^.

The four invasive plants considered here include *R. cathartica*, *F. alnus*, *A. petiolata*, and *R. multiflora*. All four of these species are categorized as Restricted by the Minnesota Department of Agriculture^45^, such that it is prohibited to import, sell, or transport propagating parts of these plants in the state. These plants encompass a range of growth habits and habitat preferences spanning a variety of forest types, forest gaps, forest and woodland edges, and managed or developed land. Previous work examining these four species illustrates a range of reported concerns from natural resource and agricultural professionals and forest owners that span conservation, economic, and recreational considerations^46^.

### Data acquisition and processing

Invasive plant location data were obtained for Minnesota and all surrounding states (Wisconsin, Iowa, South Dakota, and North Dakota) from two primary sources: the Early Detection & Distribution Mapping System^22,47^ and the USDA Forest Service’s Forest Inventory and Analysis (FIA) database^48^. Because EDDMapS is a database open to submissions via the web, it contains data from a broad range of sources including reports of invasive plant presence from federal, state, and local agencies, non-governmental organizations, and private organizations and individuals. This array of contributions from different sources results in a relatively large number of reported locations, but the nature of the data means that the locations are not systematic or free of spatial bias. Plant location data obtained from the FIA database, in contrast, are known to have been collected in a systematic fashion^48^ and are generally free of spatial bias.

Location data were processed to remove incomplete records, unverified records (EDDMapS), and duplicate points. To mitigate the impact of spatial bias as a result of non-systematic sampling in the EDDMapS data, we subsampled the location data using a 900 m^2^ grid; subsampling was completed using the gridSample() function in the *dismo* R package^49^, allowing for one sample per grid cell. Ameliorating the effects of spatial bias in sample locations is desirable because it can result in a bias in predictor space, which can affect model accuracy and transferability^50,51^. Additional data cleaning was performed by asking local professionals and experts to verify spatially isolated plant location reports. Final occurrence counts for each species and data set are listed in Table 1; spatial data are presented in Supplemental Figs. 1–4.

**Table 1:** Performance metrics for models predicting current suitable habitat using varying data sources for four invasive plant species in Minnesota, USA.

Species	Data source	*n*	AUC	Cor	*κ*	BA	PPV
*Rhamnus cathartica*	Public	2,263	0.971	0.880	0.873	0.937	0.960
*Rhamnus cathartica*	Pub + Prv	2,509	0.979	0.879	0.848	0.926	0.962
*Rhamnus cathartica*	All	2,746	0.986	0.897	0.889	0.945	0.955
*Alliaria petiolata*	Public	469	0.973	0.871	0.832	0.917	0.953
*Alliaria petiolata*	Pub + Prv	811	0.976	0.866	0.875	0.938	0.911
*Alliaria petiolata*	All	1,082	0.969	0.853	0.843	0.921	0.890
*Frangula alnus*	Public	270	0.987	0.902	0.800	0.901	0.872
*Frangula alnus*	Pub + Prv	410	0.930	0.746	0.785	0.893	0.905
*Frangula alnus*	All	445	0.948	0.796	0.796	0.897	0.850
*Rosa multiflora*	Public	138	0.994	0.941	0.899	0.947	0.936
*Rosa multiflora*	Pub + Prv	199	0.968	0.871	0.898	0.950	0.968
*Rosa multiflora*	All	208	0.998	0.941	0.952	0.976	0.969

*AUC* area under the receiver operating characteristic (ROC) curve, *cor* correlation coefficient, *κ* Cohen's kappa, *BA* balanced accuracy ((sensitivity + specificity) / 2), *PPV* positive predictive value. AUC and cor are threshold-independent, while κ, BA, and PPV are threshold-dependent metrics of performance.

We acquired environmental data for Minnesota, Wisconsin, Iowa, South Dakota, and North Dakota that spanned a number of attributes, including climate, soils, and topography. Climate data describing contemporary conditions were obtained from WorldClim (v 1.4) in the form of 30-year climate normals^52^. Future climate data were obtained from WorldClim for multi-decadal means centered on 2050 and 2070 for two different greenhouse gas emissions scenarios, as described by the Intergovernmental Panel on Climate Change (IPCC) working group: Representative Concentration Pathways (RCP) 6.0 and 8.5. RCP 6.0 represents a ‘stabilization’ emissions pathway in which the radiative forcing as a result of greenhouse gas emissions stabilizes after 2,100, while RCP8.5 represents a ‘rising radiative forcing’ pathway where radiative forcing as a result of greenhouse gas emissions continues to increase^42^. In order to expand the generalization of the analyses, we constructed models for these climate scenarios under two different climate models: the CMIP5 HadGEM2-ES Earth System (hereafter ‘HadGEM’) and the Community Climate System (CCSM4, hereafter ‘CCSM’)^52–54^. These two climate models were chosen because they both see wide application and have generally been found to perform well for North America^55,56^. All climate data were obtained in the form of 19 bioclimatic variables, as described by Hijmans et al. (2005)^52^.

Soils data were obtained in the form of STATSGO soil survey data from the Natural Resources Conservation Service (NRCS) web soil survey^57^. Specific soils data acquired included soil particle size, soil drainage class, and soil taxonomic group. Elevation data were obtained from the National Elevation Dataset (NED)^58^. All predictor data were projected to a spatial resolution of 900 m^2^; where necessary, continuous predictors were resampled using bilinear interpolation and categorical variables were resampled using a nearest-neighbor approach.

### Distribution modeling

Distribution models were created using a random forests (RF) approach^59^, as implemented in the *randomForest* package in R (version 4.6–14; R version 3.6.1)^60,61^. Pseudo absences were generated for each species according to the recommendations outlined in Barbet-Massin et al. (2012)^62^. Prior to analysis, model selection was conducted for each species using the approach described by Murphy et al. (2010)^63^ and as implemented by the modelSel() function of the *rfUtilities* package (version 2.1-5)^64^. This approach allows for parsimonious model and variable selection without sacrificing model performance. We used a row standardization approach to model selection with a parsimony factor (allowable error for competing models) of 0.05. This process allowed us to eliminate 11 variables from our analyses, as they either had consistently low importance or demonstrated high multicollinearity with more important variables. Variables used for model training therefore included: elevation, soil particle size, soil drainage, soil taxonomic group, mean annual temperature, mean annual precipitation, mean temperature of the coldest quarter, mean temperature of the warmest quarter, mean precipitation of the driest quarter, mean precipitation of the wettest quarter, precipitation seasonality (coefficient of variation of precipitation), and temperature seasonality (standard deviation * 100).

While our primary area of interest was Minnesota, models were trained on a broad five-state region consisting of data from Minnesota, Wisconsin, Iowa, South Dakota, and North Dakota to avoid niche truncation and provide additional training data. Model performance was evaluated using the internal RF performance metrics produced using out-of-bag sampling, as well as a 70:30 training:testing data split to minimize data leakage. Performance was assessed using threshold-dependent metrics, including Cohen’s kappa (κ), balanced accuracy, and positive predictive value, and threshold-independent metrics, including the area under the receiver operating characteristic curve (AUC) and the correlation coefficient. Variable importance was assessed using the mean decrease in node impurity, which is a relative measure of how well trees in the random forest split the data. Continuous and binary distribution estimate maps were produced for Minnesota for each species; binary maps were created using a threshold based on the maximization of model specificity and sensitivity. Climate change projections using the HadGEM and CCSM climate projections were created for each distribution model under both the RCP 6.0 and 8.5 pathways for the 2050s and 2070s.

To assess the impact of modeling invasive plant distributions using data from different sources, we created separate sets of models for each species using: (1) only data from public (i.e., government) sources, i.e., the FIA database, and federal, state, local, and tribal government sources entered into EDDMapS, (2) data from both public and private sources, i.e., the previous plus data from non-governmental organizations, the general public, volunteer groups, and other private sources entered into EDDMapS, and (3) all available data, i.e., data from the previous plus all data without an explicitly-defined source entered into EDDMapS.

The impact of climate change on each species’ predicted distribution over time was assessed using a mixed-effects model with the predicted distribution area as the response and the climate scenario as a fixed-effect variable and data source-set as a random factor. The impact of data set source on predicted distribution was assessed similarly, with predicted distribution area as the response variable and data set source as a fixed-effect variable and climate model as a random factor. Mixed-effects models were computed using the *lme4* package in R (version 1.1–21)^65^. The impact of data set source on model performance was assessed for each species using linear models. To visualize the impact of climate change scenario and data set source on model estimates, density plots were created for each model. To assess uncertainty associated with data source (public, public and private, all available data), we computed standard deviation rasters for modeled current distributions as well as each climate change scenario and timepoint.

## Results

**Figure 1:**
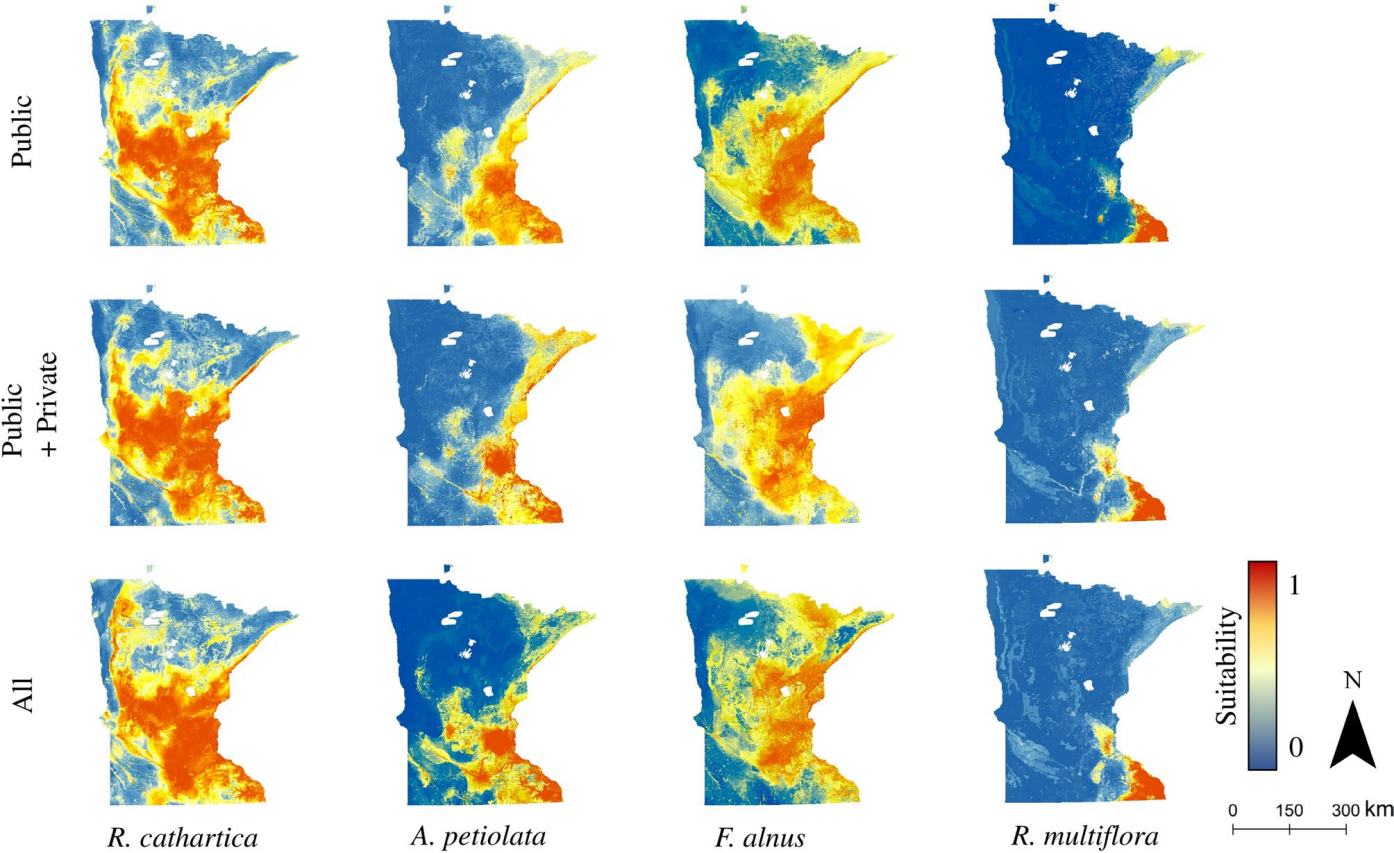
Distribution model (Random Forest) output for all four species, across data source sets. Current climate conditions, based on 30-year normals (Hijmans et al. 2005). Public: models trained based on occurrence data obtained from public (i.e., governmental) sources; Public + Private: models trained on data obtained from public as well as private sources; All: models trained on all available data, regardless of reported source.

**Figure 2:**
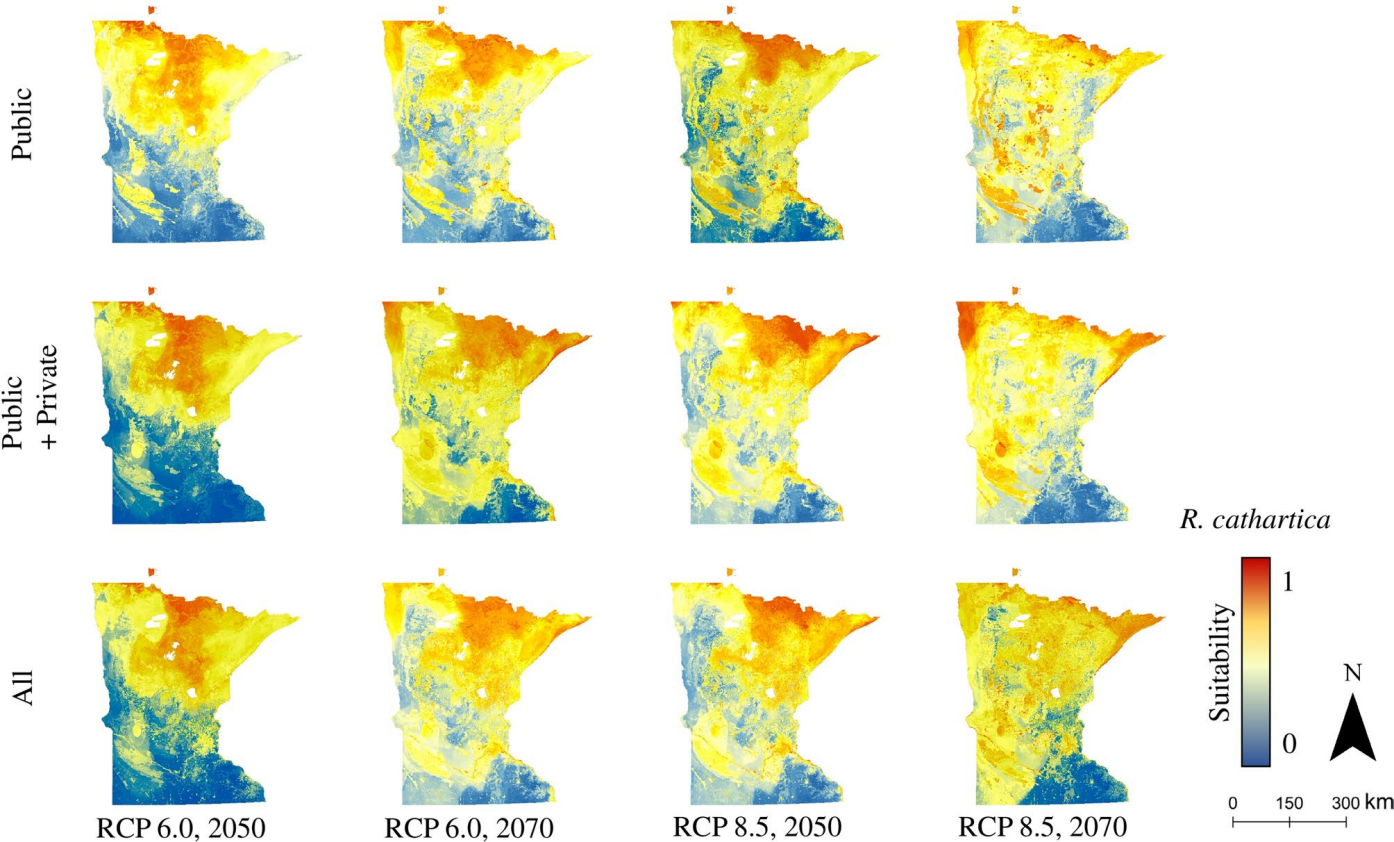
*R. cathartica* distribution model (Random Forest) output across data source sets, for future climate conditions (RCP 6.0 and 8.5, 2050s and 2070s) under the HadGEM climate model. Public: models trained based on occurrence data obtained from public (i.e., governmental) sources; Public + Private: models trained on data obtained from public as well as private sources; All: models trained on all available data, regardless of reported source.

**Figure 3:**
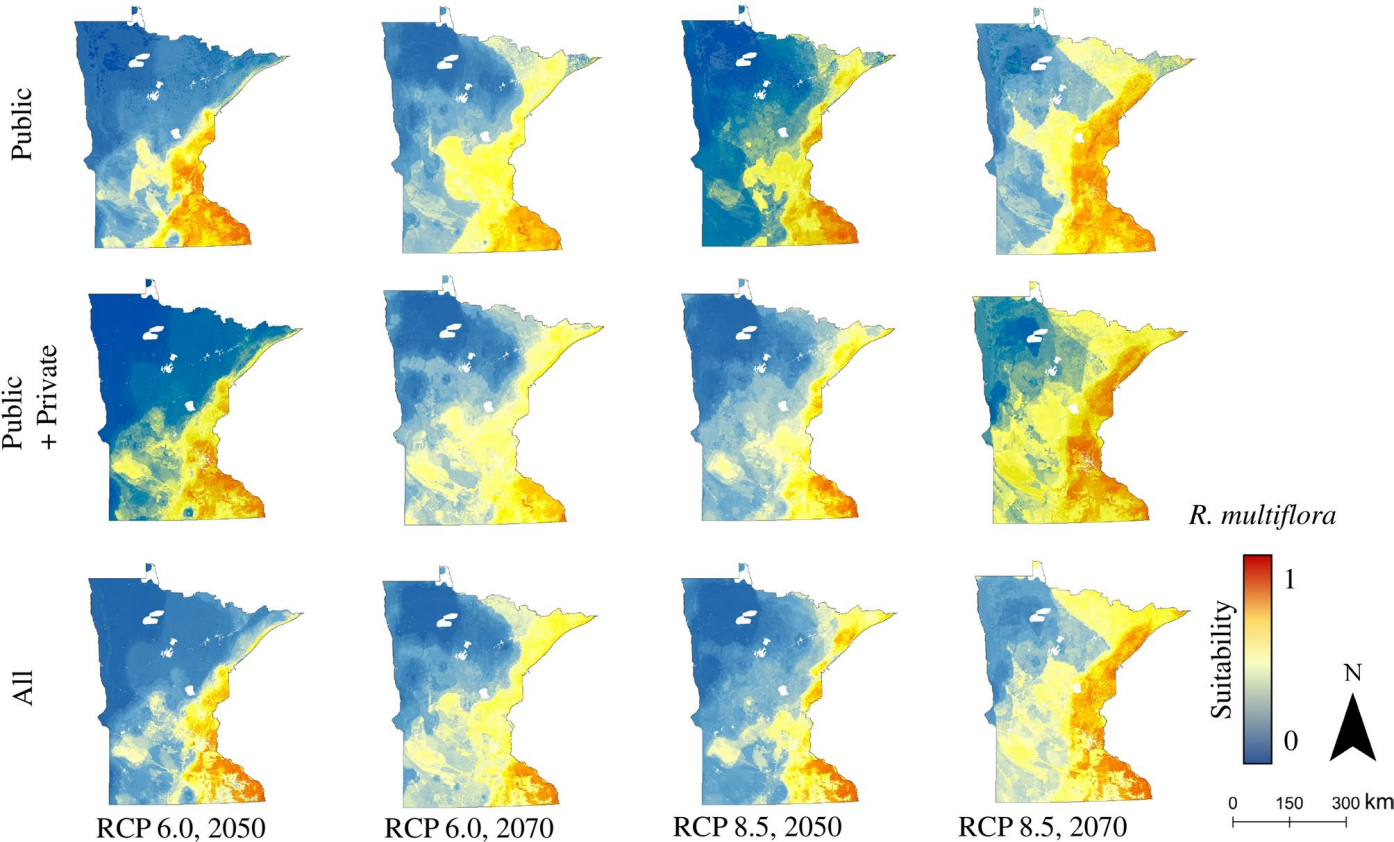
*R. multiflora* distribution model (Random Forest) output across data source sets, for future climate conditions (RCP 6.0 and 8.5, 2050s and 2070s) under the HadGEM climate model. Public: models trained based on occurrence data obtained from public (i.e., governmental) sources; Public + Private: models trained on data obtained from public as well as private sources; All: models trained on all available data, regardless of reported source.

A total of 27 distribution models were created for each species (Figs. 1, 2, 3, Supplemental Figs. 5–10), encompassing current and future climate scenarios across two climate models and a number of different data sets. In general, models performed well (Table 1), with values of κ > 0.75 and AUC > 0.90. Model data set source had no significant impact on performance in general (χ^2^ = 2.03, df 2, P = 0.362). *Frangula alnus*, which had 140 privately-reported plant locations (out of *n* = 445, Table 1) demonstrated the lowest overall κ and correlation coefficients in the public and public + private data source models, but a similar pattern was not found in the performance results for *A. petiolata*, which also demonstrated a fairly large proportion of privately-reported plant locations (342 out of *n* = 1,082, Table 1).

Climatic variables were consistently among the most important predictors for each species across models (Table 2). Precipitation variables in particular were consistently important for each species, especially annual precipitation and precipitation of the driest month. Temperature variables were consistently important for *R. cathartica, F. alnus,* and *R. multiflora*, but not for *A. petiolata*. Non-climate variables, particularly soil particle size and elevation, were more important for *A. petiolata* relative to the other species.

**Table 2:** Summary of the top four most important variables for the random forest model of each species, as determined by the mean decrease in node impurity from splitting on each variable.

Species	Public		Public + Private		All	
	Variable	Importance	Variable	Importance	Variable	Importance
*R. cathartica*	Precip. Driest Month	154.3	Annual Precip	174.2	Annual Precip	196.8
	Annual Precip	129.2	Precip. Driest Month	158.9	Precip. Driest Month	142.6
	Mean Temp. CQ	127.5	Annual Mean Temp	153.3	Annual Mean Temp	130.7
	Annual Mean Temp	121.6	Mean Temp. WQ	109.5	Mean Temp. WQ	124.3
*A. petiolata*	Precip. Seasonality	34.6	Precip. Seasonality	77.4	Precip. Seasonality	101.8
	Annual Precip	27.5	Annual Precip	61.7	Precip. Driest Month	83.2
	Precip. Driest Month	27.4	Precip. Driest Month	48.5	Annual Precip	64.8
	Soil Particle Size	27.2	Precip. Wettest Month	39.0	Elevation	51.8
*F. alnus*	Annual Precip	15.1	Annual Precip	30.8	Annual Precip	34.0
	Annual Mean Temp	11.8	Annual Mean Temp	23.5	Annual Mean Temp	25.7
	Precip. Driest Month	11.8	Precip. Driest Month	20.2	Precip. Driest Month	19.6
	Mean Temp. CQ	11.5	Mean Temp. CQ	18.6	Mean Temp. CQ	17.7
*R. multiflora*	Annual Precip	15.1	Precip. Seasonality	19.6	Annual Precip	20.3
	Precip. Driest Month	9.9	Precip. Driest Month	18.2	Precip. Driest Month	19.7
	Precip. Seasonality	9.8	Annual Precip	15.9	Precip. Seasonality	17.4
	Annual Mean Temp	7.9	Mean Temp. CQ	8.8	Annual Mean Temp	8.9

Importance values are relative and are not comparable between species.

**Figure 4:**
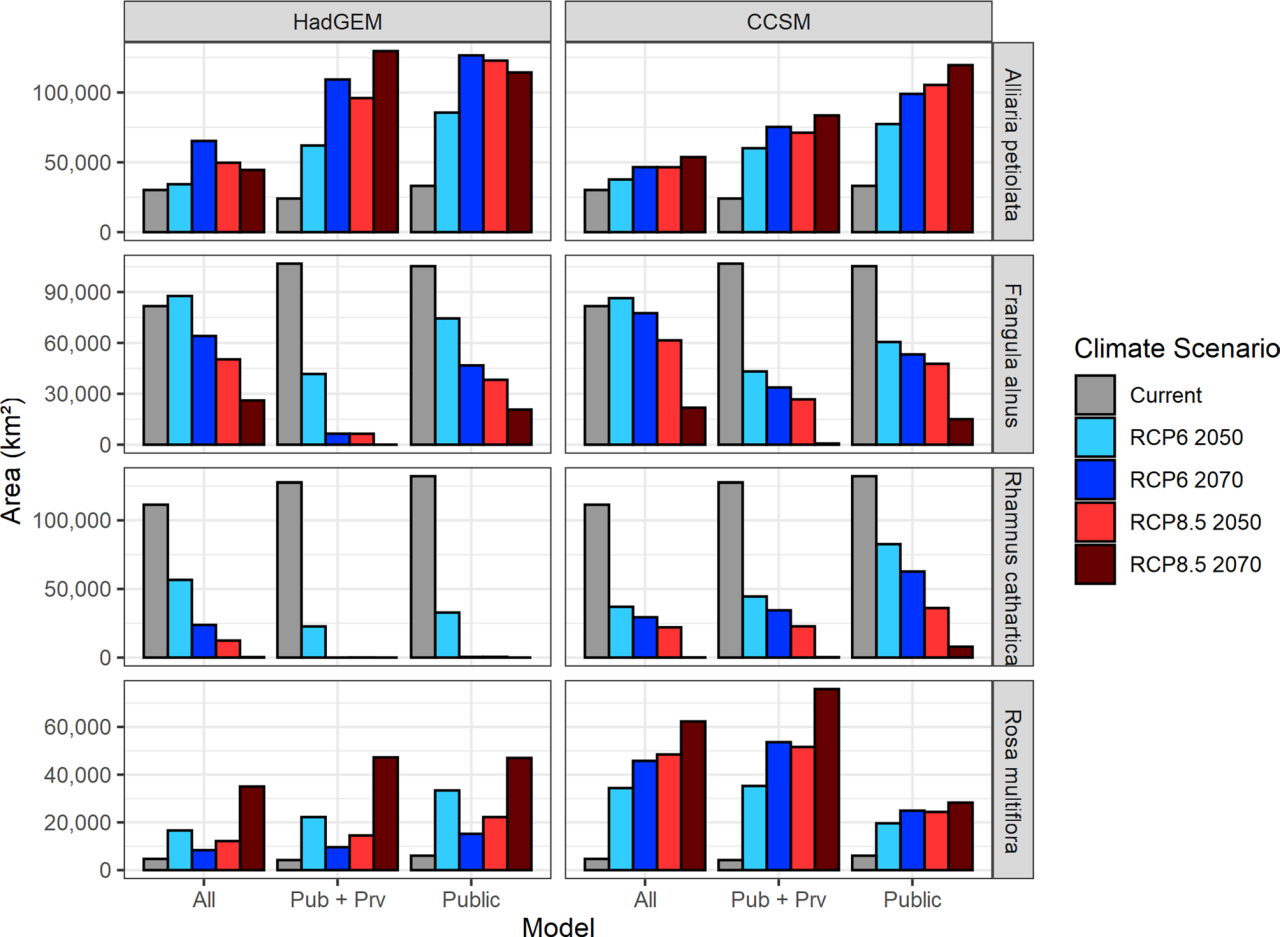
Mean area (km^2^) estimates for current and future projections of suitable habitat, across varying climate scenarios using two climate models (HadGEM and CCSM) and across three data sources for four invasive plant species in Minnesota, USA. Public: models trained based on occurrence data obtained from public (i.e., governmental) sources; Pub + Priv: models trained on data obtained from public as well as private sources; All: models trained on all available data, regardless of reported source.

Climate change had a significant impact on the amount of suitable habitat predicted for each species under scenarios from both climate models (Fig. 4, Supplemental Table 1). For *R. cathartica* and *R. multiflora*, changes in total predicted suitable habitat under the HadGEM scenarios were significant across all scenarios and timepoints (*R. cathartica* χ^2^ = 258.92, *P* < 0.001, *R. multiflora* χ^2^ = 197.84, *P* < 0.001). For *A. petiolata* and *F. alnus*, the impact of climate scenario was significant overall (*A. petiolata* χ^2^ = 29.71, *P* < 0.001, *F. alnus* χ^2^ = 47.87, *P* < 0.001), but the largest differences were apparent in the warmer scenarios (*A. petiolata:* 2070s RCP 6.0 *t* = 4.57, RCP 8.5 *t* = 4.30, *F. alnus*: 2070s RCP 6.0 *t* = − 4.41, RCP 8.5 *t* = − 6.17). Both buckthorn species were projected to lose suitable habitat under both RCPs through the 2070s, while *A. petiolata* and *R. multiflora* are projected to gain suitable habitat (Fig. 4). Changes in total predicted suitable habitat were significant across all scenarios and timepoints for all species under the CCSM climate models (*A. petiolata* χ^2^ = 53.38, *P* < 0.001, *F. alnus* χ^2^ = 78.76, *P* < 0.001, *R. cathartica* χ^2^ = 465.2, *P* < 0.001, *R. multiflora* χ^2^ = 72.16, *P* < 0.001).

Area of predicted suitable habitat (Fig. 4) was significantly affected by model data set source for three of the four species under the HadGEM model: *A. petiolata* (χ^2^ = 19.98, df 2, *P* < 0.001)*, F. alnus* (χ^2^ = 9.51, df 2, *P* = 0.009)*,* and *R. multiflora* (χ^2^ = 16.83, df 2, *P* < 0.001). Predicted suitable habitat was highest using the public-only data set for *A. petiolata* and *R. multiflora*, except for the 2070 RCP 8.5 scenario, where public + privately sourced data demonstrated slightly larger area estimates for both species (Fig. 4). The *F. alnus* model constructed using the public + privately sourced dataset resulted in lower predicted suitable habitat area than both the publicly sourced dataset and the 'all available' dataset. Models constructed using all available data yielded the largest area estimates for *F. alnus* and *R. cathartica* in all future climate change scenarios (Fig. 4). Under the CCSM model area of predicted suitable habitat was significantly affected by data set source for all species (*A. petiolata* χ^2^ = 37.5, *P* < 0.001, *F. alnus* χ^2^ = 8.08, *P* = 0.017, *R. cathartica* χ^2^ = 25.07, *P* < 0.001, *R. multiflora* χ^2^ = 21.41, *P* < 0.001). Models built using the public-only data set demonstrated the highest predicted suitable habitat for *A. petiolata* and *R. cathartica* under the CCSM climate model*,* while models built with all available data had the highest area estimates for *F. alnus*. Despite higher suitable habitat values for *R. cathartica* in the 2050s time points, models built with the public-only dataset still ended up near zero in the warmest climate scenario (RCP 8.5, 2070s), similar to the models built with additional data. The CCSM-based models built with all data and public + private data were similar for *R. multiflora*, except in the RCP 8.5 scenario at 2070, where public + private was higher (Fig. 4).

**Figure 5:**
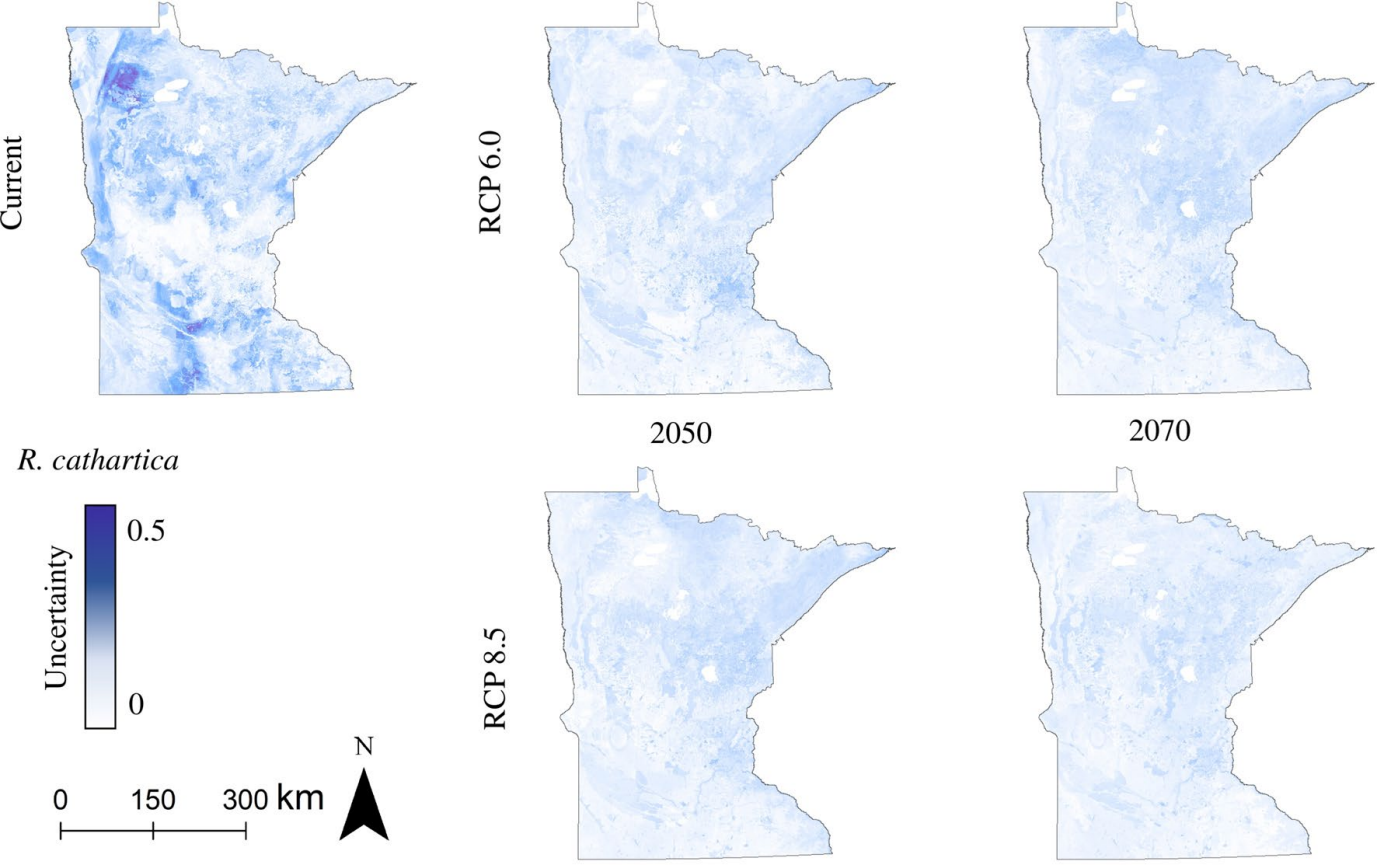
Distribution model uncertainty attributable to data set source, *R. cathartica*. Uncertainty is quantified as the standard deviation between rasters of different data set sources (Public, Public + Private, All) for all climate scenarios using the HadGEM climate model.

**Figure 6:**
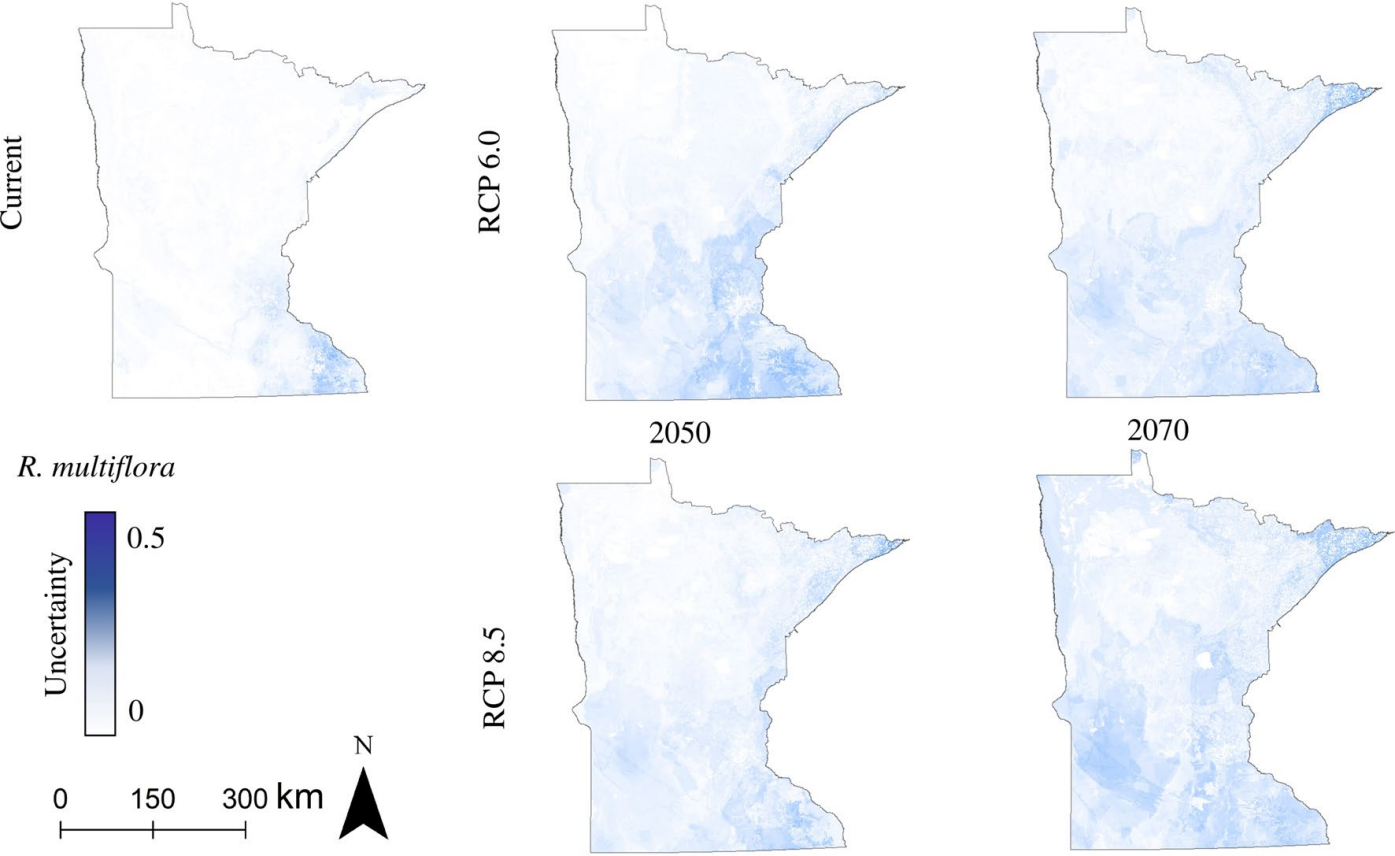
Distribution model uncertainty attributable to data set source, *R. multiflora*. Uncertainty is quantified as the standard deviation between rasters of different data set sources (Public, Public + Private, All) for all climate scenarios using the HadGEM climate model.

For *A. petiolata, F. alnus,* and *R. cathartica,* the uncertainty attributable to data set source was highest for models constructed with current climate conditions (Fig. 5, Supplemental Figs. 11–15). For *R. multiflora*, the species with the smallest current distribution in Minnesota, uncertainty was more widespread in future predictions, both in terms of spatial distribution (Fig. 6, Supplemental Fig. 16) and overall value distribution (Supplemental Figs. 20 & 24).

## Discussion

The current and future distribution estimates produced for each of the four invasive plant species in this study provide a synoptic view of potential areas of concern for these species over time, while also highlighting the utility of large, semi-open databases such as EDDMapS. The distribution models produced here are, to our knowledge, the first detailed spatially-continuous estimates of both current and (climate-mediated) future habitat suitability for these four species in the Minnesota region. Previous spatially-explicit analyses for these species have focused on contemporary climate conditions and have either been broad in scope or focused on other regions. Broad-scale distributional and habitat analyses have previously been conducted for *A. petiolata*^66–68^ and *R. multiflora*^69^, while local-scale risk and spread models have been constructed for *A. petiolata*^70^ and *R. cathartica*^71^for some locales (the Upper Peninsula of Michigan for *A. petiola* and Waterloo, Ontario for *R. cathartica*).

Model projections suggest that climate change will have a significant impact on the amount of suitable habitat within Minnesota for each of the four invasive plant species studied (Fig. 4). General trends (i.e., increasing or decreasing) are similar across both HadGEM and CCSM projections. Both buckthorn species are projected to lose large amounts of suitable habitat in the warmest climate scenarios (e.g., RCP 8.5, and 2050s RCP 6.0). While there is relatively little research regarding the direct and indirect impacts of climate change on *F. alnus* physiology, growth, and recruitment, there is a growing body of such literature for *R. cathartica*. Experimental work from Reich et al. (2018)^72^ suggest warming of + 3.4 °C above current temperatures may increase net photosynthesis when soil moisture levels are high. Other work has found that both flooding and drought have significant negative impacts on *R. cathartica* photosynthesis^12^. The importance of moisture indicated by these studies tracks with our constructed models: we found precipitation variables to be consistently among the most important predictors in each *R. cathartica* model (Table 2), particularly annual precipitation and precipitation of the driest month. Additional work suggests that warming temperatures will have varying impacts on *R. cathartica*; Wright et al. (2018)^73^ found a negative relationship between seedling emergence rate and warming temperatures, while Fisichelli et al. (2014)^74^ found a positive or nonexistent relationship, depending on whether leaf litter was present. Overall, the potential impacts of a changing climate on *R. cathartica* seem complex. Based on current climate conditions and the distribution of this species in the upper Midwest region, our models suggest that habitat suitability will likely decline by the late twenty-first century in the two climate change RCPs considered here.

In contrast to the buckthorn species, our results suggest that *A. petiolata* and *R. multiflora* are predicted to gain suitable habitat within Minnesota (Fig. 4). Both of these species are distributed much more abundantly in the southern region of the model training area (i.e., Iowa, southern Wisconsin, southern Minnesota) and beyond; *A. petiolata* is common in southern Illinois, Kentucky, Tennessee, and is found as far south as Georgia, while *R. multiflora* is widely distributed across the eastern United States, from the southeastern corner of Minnesota to the Gulf shore of Texas^22^. The wider distribution of these two species relative to the buckthorn species suggests a broader niche breadth^75,76^. Species with broad niche breadths and demonstrated ability to thrive in significantly warmer climates may be more likely to respond positively to a warming global climate^77–79^, particularly at the leading edge.

While data composition did not have a significant impact on model performance, it did affect model area estimates. The area of predicted suitable habitat varied particularly under the warmest climate conditions (e.g., 2070s for both RCP 6.0 and 8.5, Fig. 4). For example, among the *A. petiolata* models, the difference between data set source was relatively smaller under current climate conditions (maximum 33,108 km^2^, minimum 24,055 km^2^) compared to the warmest scenario (maximum 129,630 km^2^, minimum 44,534 km^2^) (Fig. 4). This may reflect the occurrence data used to train models for this species; private and other non-public data make up a large proportion (~ 40%) of all available occurrence points for *A. petiolata* (Table 1, Supplemental Fig. 2). Private and other non-public data make up a similarly large portion (~ 44%) of occurrence data for *R. multiflora*, which also demonstrates apparent differences between models constructed using public data only and those trained using public + private or all available data (Fig. 4). These differences may be due to the large proportion of non-public occurrence data for these species having a different distribution pattern or a varying amount of sampling bias relative to public data (Supplemental Figs. 2 and 4). These results may suggest that differences in model predictions which may be relatively minor under contemporary environmental conditions could become more pronounced if projections are made for changing environmental conditions. This can be a concern because, as demonstrated here, there may be species for which much of the available spatial data is scattered across different sources, some of which may be from public agencies, ecological professionals, or herbaria, but some may come from volunteer citizen groups, private companies, the general public, or may even be listed in a database but have unattributed sourcing. We find that models constructed with different subsets of these data can yield the same broad, overall result (i.e., increase or decrease in predicted suitable habitat over time and climate scenarios), though the magnitude and specific areal predictions may vary (Figs. 4, 5 and 6).

The models produced here performed well by all available metrics (Table 1), demonstrating good capacity for prediction^80^. There are, however, limitations inherent to species distribution modeling and making projections under future climate scenarios. While distribution modeling is generally performed using data from the realized niche of a species^39^, factors important to shaping the realized niche, particularly biotic interactions and population dynamics, are not considered in standard species distribution modeling approaches. Moreover, because the four species we analyzed are exotic invasives, the spatial data available for model construction may not reflect equilibrium range limits^81,82^; there is, however, a growing set of literature demonstrating the utility of distribution modeling approaches for invasive species ecology and management despite this concern^37,38,83^. Another important caveat is that this study models the distribution of these four invasive plants using regional occurrence data and does not incorporate data from the species’ native range or non-regional invaded range. This was done because Minnesota represents a leading edge of the invasive range for each of these species, and in working with local managers, we were interested in identifying habitats similar to those already invaded in Minnesota. We were particularly interested in locally-relevant habitats following several recent studies demonstrating that local adaptations and niche shifts may be more common than previously thought among invasive species^38,84,85^. A limitation of this approach, however, is that if these species exhibit realized niche conservatism, we may be underestimating total suitable habitat.

The suitable habitat predictions for each of the four species studied here provide additional context for future conservation and invasive species management in Minnesota. The buckthorn species considered here—*R. cathartica* in particular—are among the most abundant and noticeable woody invaders in Minnesota forests, and have been linked to substantial changes in forest regeneration and nutrient cycling^11,15^, loss of biodiversity and ecosystem function^11,15,86–88^, and hosting agricultural pests, particularly soybean aphids (*Aphis glycines*)^89^. The loss of suitable habitat in Minnesota as the climate warms, as predicted by our results (Figs. 2, 4, and Supplemental Figs. 6–7, 9), may eventually contribute to a gradual decline of buckthorn across the state.

As one of the few herbaceous plant species able to successfully invade forest understory plant communities in the U.S.^23^, *A. petiolata* is disruptive to both understory communities and forest regeneration by impacting local mycorrhizal fungi populations^87,90,91^, altering tree seedling abundance and composition^27^, and competing with native understory vegetation^24–26^. The moderate to large increase in suitable habitat across the state predicted by our results (Fig. 4, Supplemental Figs. 5, 8) suggests that *A. petiolata* will continue to be a concern into the future as the climate warms. The potential impacts of further *A. petiolata* invasion, particularly on forest regeneration and mycorrhizal communities, may be further exacerbated if patterns of forest disturbance and fragmentation increase^23,66,92–94^ alongside warming temperatures and shifting precipitation patterns, though further research is needed, as there is a lack of data examining the interactive impacts of potential climate and land use change on *A. petiolata*.

Similar to *A. petiolata, R. multiflora* is rare among invasive plants in that it can be found in intact understories within closed-canopy forests^28,29^, though it is more frequently found in forested areas with relatively low basal area, forest gaps, and edges^95–97^. The fairly large increase in suitable habitat our models predict for *R. multiflora* under different climate change scenarios (Figs. 3, 4) is concerning because the species frequently forms dense thickets along forest edges and in forest gaps, inhibiting the growth of other species^30^. Outside of forest-related concerns, recent research by Adalsteinsson et al. (2018)^98^ suggests that areas invaded by *R. multiflora* are associated with an increased prevalence of *Borrelia burgdorferi*, the bacterial source of Lyme disease, in black-legged ticks (*Ixodes scapularis*); though, the dense vegetation structure of *R. multiflora*-invaded stands does not necessarily make human disease risk more likely, as nymphs in dense invaded stands are hypothesized to feed primarily on smaller-bodied hosts such as mice. The predicted growth of suitable habitat for *R. multiflora* in northern Minnesota as time and climate progress, particularly along the shore of Lake Superior, is likely to be of concern in the future, as there is an abundance of forestland fragmented by recreational trails, roads, and harvest gaps in the region.

Here, we present a series of predictions outlining the potential current and future distributions of four forest plant invaders in Minnesota, as well as an assessment regarding the impact of two climate change scenarios across two different climate models and the importance of input data source. Results suggest a potential loss of suitable habitat in Minnesota for both buckthorn species under the climate change scenarios considered here, and a gain for *R. multiflora* and *A. petiolata*. Indeed, our results suggest that climate change affects the area and spatial distribution of predictions, and that differences between models constructed with different input data become more pronounced over time and in warmer scenarios. The predictions produced as part of this work are a function of the availability of data for each species, and an increase in reporting effort would likely improve the accuracy and utility of such models; the continued use of tools such as EDDMapS and of citizen scientist data collection in general^99,100^ are promising in this respect. The continued development and interconnectivity of databases and data-driven tools is an important factor in tackling the ongoing challenge of invasive species in forest management and conservation. Indeed, the future of forest management and conservation is faced with an abundance of uncertainty, whether from climate change, invasive plants, anthropogenic activity, or otherwise^101–104^. Research that seeks to shed light on these uncertainties, such as the analyses performed here, is an important component of the forest ecology and management toolbox.

## Supplementary information

Supplementary file1
