# Supplementary material for: Assessing the current and potential future distribution of four invasive forest plants in Minnesota, U.S.A., using mixed sources of data
Source: Sci Rep. 2020 Jul 29;10:12738. doi: 10.1038/s41598-020-69539-1 (PMC7392769; doi:10.1038/s41598-020-69539-1)
Supplement: Supplementary file 1 — Supplementary file1 [file 41598_2020_69539_MOESM1_ESM.pdf]

Assessing the Current and Potential Future Distribution of Four Invasive Forest Plants in Minnesota, U.S.A., Using Mixed Sources of Data

JASON R. REINHARDT*, *Department of Forest Resources, University of Minnesota, Minneapolis, MN*

MATTHEW B. RUSSELL, *Department of Forest Resources, University of Minnesota, Minneapolis, MN*

SENAIT SENAY, *Department of Plant Pathology, University of Minnesota, Minneapolis, MN*

WILLIAM LAZARUS, *Department of Applied Economics, University of Minnesota, Minneapolis, MN*

* Corresponding author: reinh215@umn.edu

Supplemental Materials

Supplemental Tables

Table 1. Mean area (km²) estimates for current and future projections of suitable habitat, across varying climate scenarios and data sources for four invasive plant species in Minnesota, USA. Values rounded to the nearest square kilometer.

			HadGEM				CCSM			
			RCP 6.0		RCP 8.5		RCP 6.0		RCP 8.5	
Species	Model	Current	2050	2070	2050	2070	2050	2070	2050	2070
R. cathartica	Public	131920	32811	466	506	0	82619	62719	36099	7916
R. cathartica	Pub + Prv	127450	22689	5	68	0	44502	34457	22757	318
R. cathartica	All	111370	56607	23787	12395	340	36991	29377	22057	117
A. petiolata	Public	33108	85582	126556	122832	114324	77394	98961	105396	119572
A. petiolata	Pub + Prv	24055	62028	109283	95984	129630	60078	75345	71212	83561
A. petiolata	All	30176	34298	65295	49694	44534	37706	46524	46487	53737
F. alnus	Public	105226	74450	46755	38245	20688	60544	53231	47687	15049
F. alnus	Pub + Prv	106744	41687	6496	6450	0	43193	33746	26732	685
F. alnus	All	81705	87662	64088	50311	26094	86393	77544	61519	21789
R. multiflora	Public	6048	33394	15214	22221	46995	19611	24908	24381	28291
R. multiflora	Pub + Prv	4210	22226	9603	14537	47240	35256	53616	51611	75860
R. multiflora	All	4702	16615	8368	12165	35024	34383	45776	48467	62293

Supplemental Figures

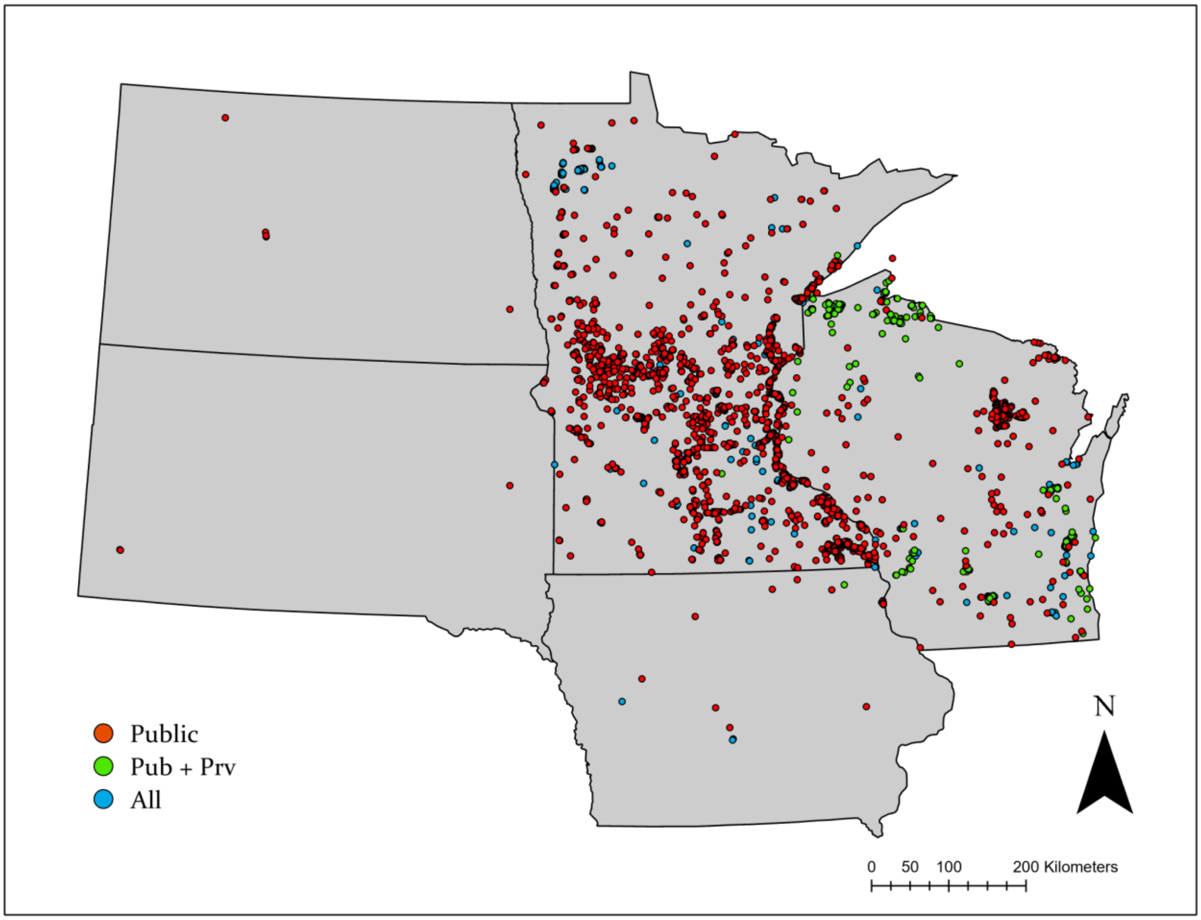

Figure 1. Distribution of model training points for *R. cathartica*. Colors correspond to data set source; the more inclusive data source sets (i.e., ‘Pub + Prv’ and ‘All’) include the points in lower set(s) (e.g., the ‘All’ data set includes all of the ‘Pub + Prv’ points plus all other available points, regardless of source). Points were obtained from EDDMapS and the USDA Forest Service’s FIA database.

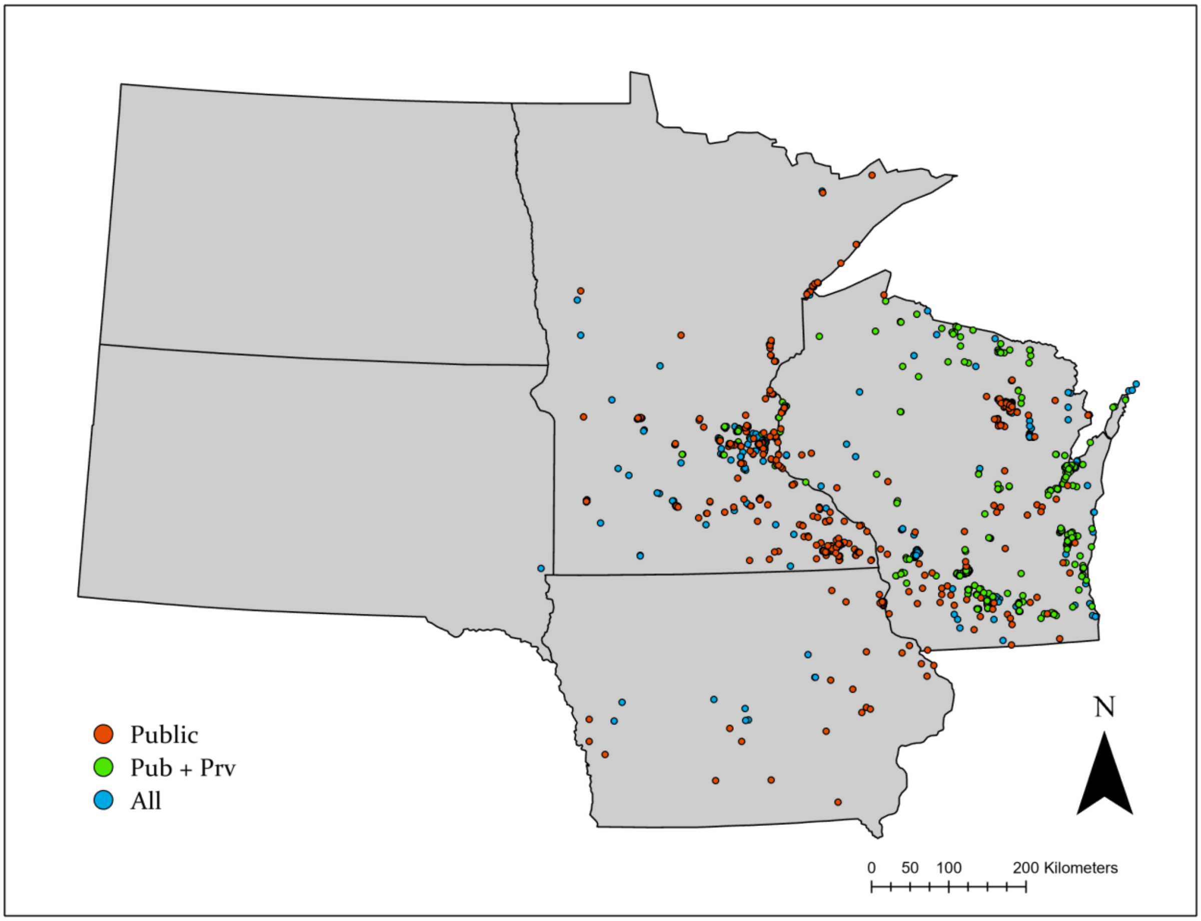

Figure 2. Distribution of model training points for *A. petiolata*. Colors correspond to data set source; the more inclusive data source sets (i.e., ‘Pub + Prv’ and ‘All’) include the points in lower set(s) (e.g., the ‘All’ data set includes all of the ‘Pub + Prv’ points plus all other available points, regardless of source). Points were obtained from EDDMapS and the USDA Forest Service’s FIA database.

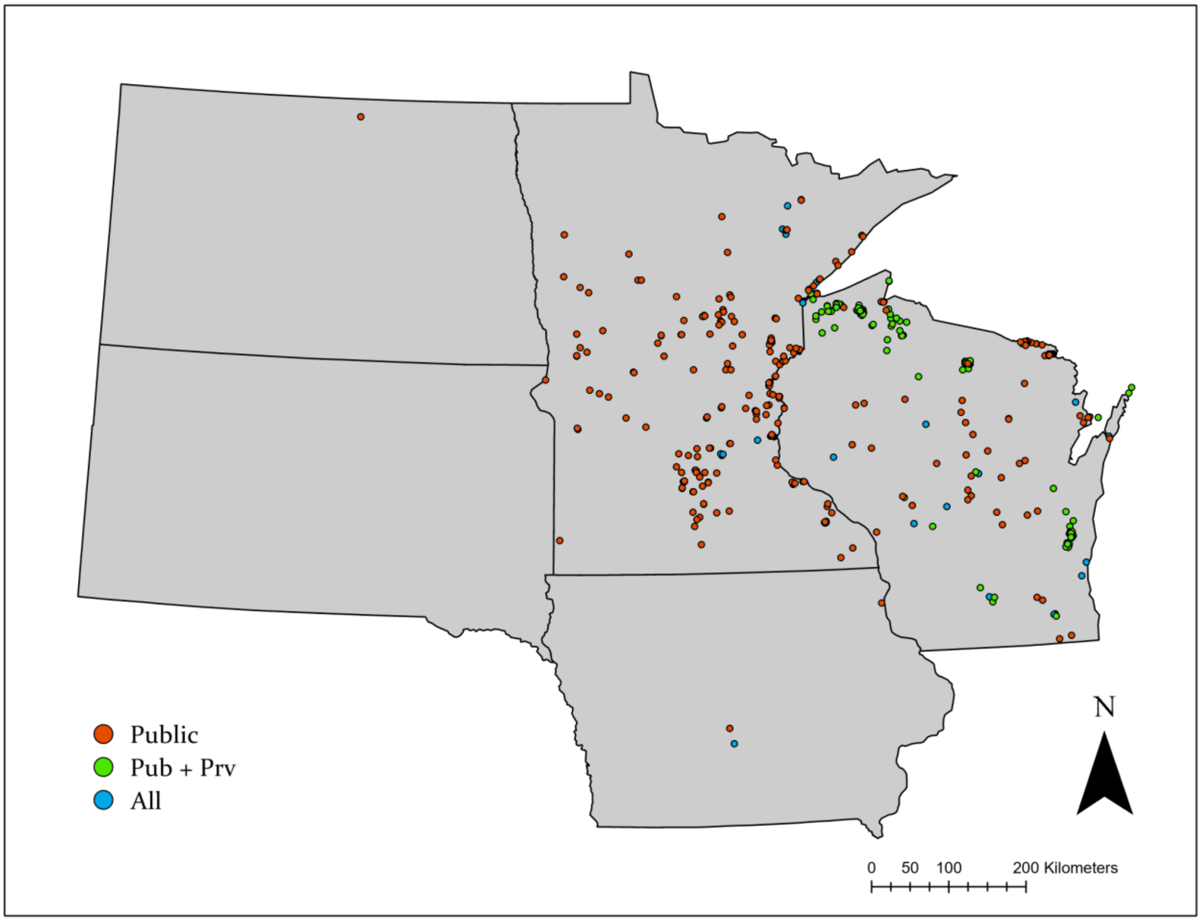

Figure 3. Distribution of model training points for *F. alnus*. Colors correspond to data set source; the more inclusive data source sets (i.e., 'Pub + Prv' and 'All') include the points in lower set(s) (e.g., the 'All' data set includes all of the 'Pub + Prv' points plus all other available points, regardless of source). Points were obtained from EDDMapS and the USDA Forest Service's FIA database.

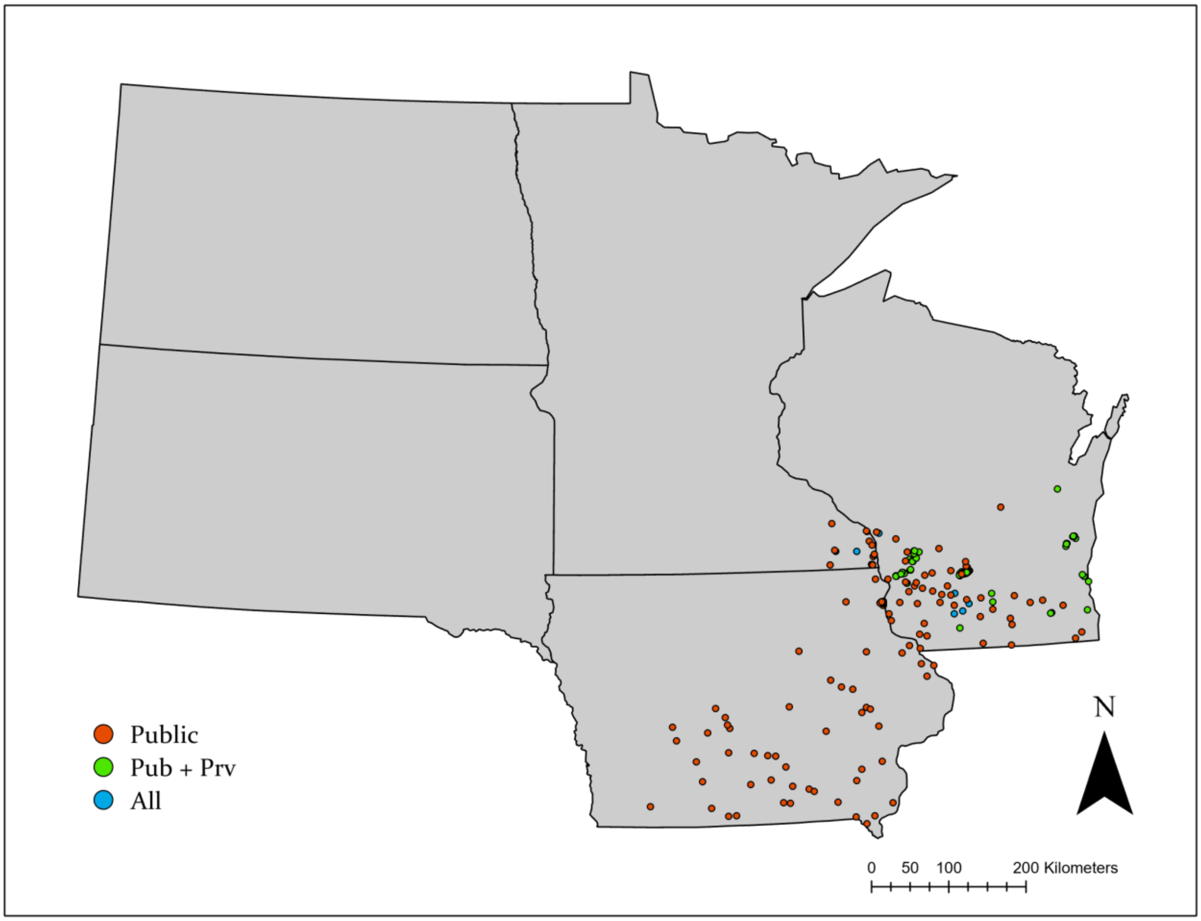

Figure 4. Distribution of model training points for *R. multiflora*. Colors correspond to data set source; the more inclusive data source sets (i.e., 'Pub + Prv' and 'All') include the points in lower set(s) (e.g., the 'All' data set includes all of the 'Pub + Prv' points plus all other available points, regardless of source). Points were obtained from EDDMapS and the USDA Forest Service's FIA database.

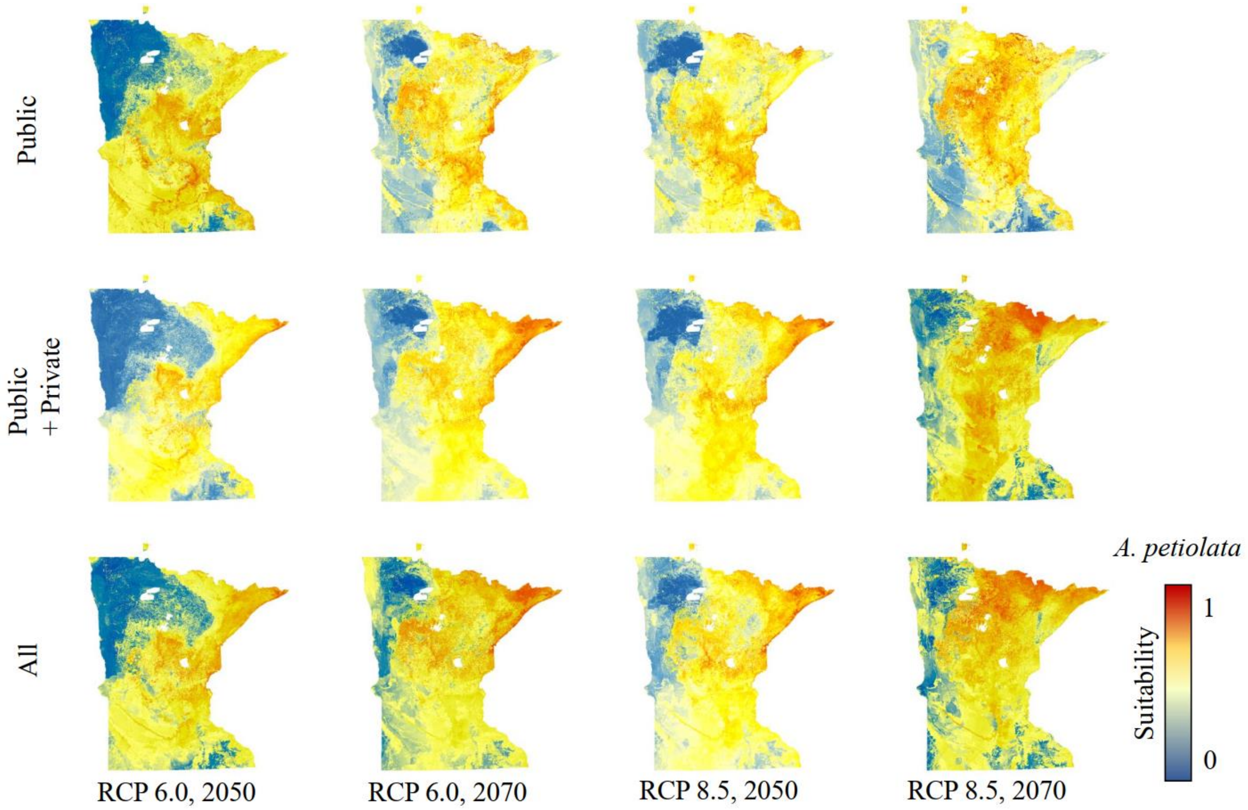

Figure 5. *A. petiolata* distribution model output across data source sets, for future climate conditions (RCP 6.0 and 8.5, 2050s and 2070s) under the HadGEM climate model.

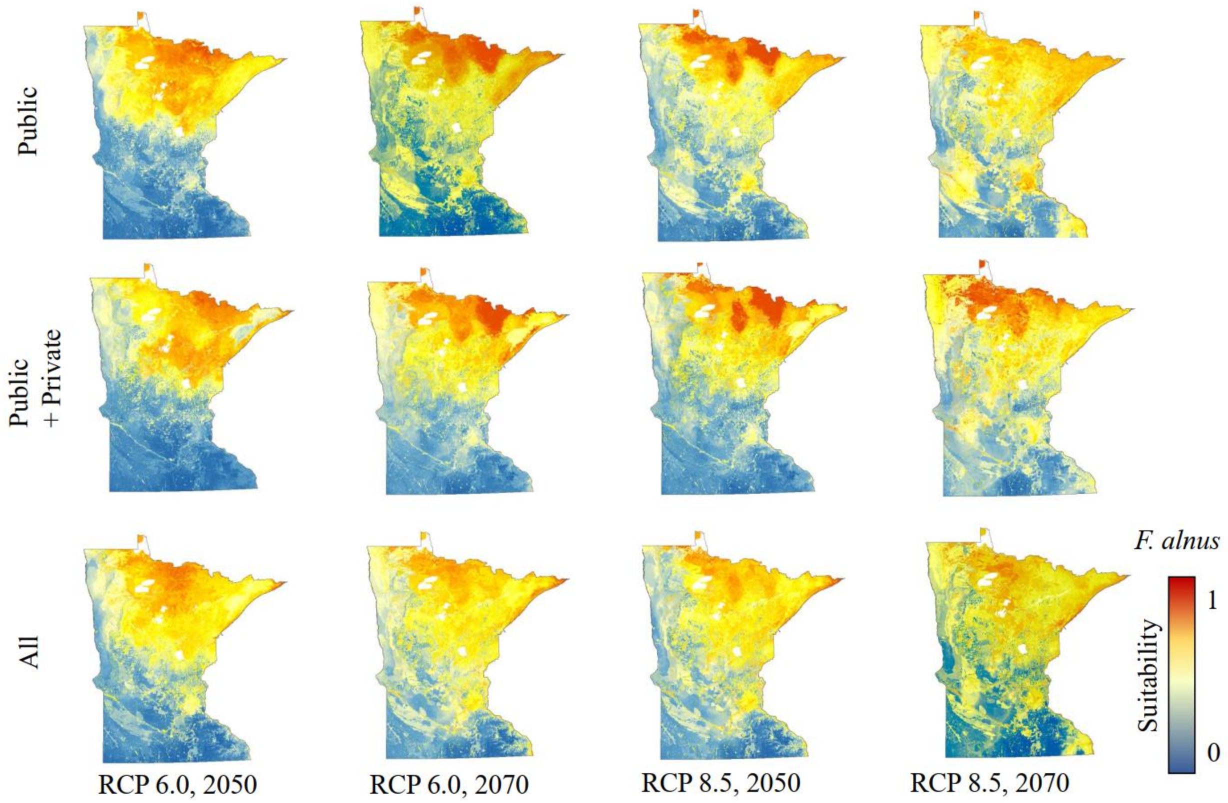

Figure 6. *F. alnus* distribution model output across data source sets, for future climate conditions (RCP 6.0 and 8.5, 2050s and 2070s) under the HadGEM climate model.

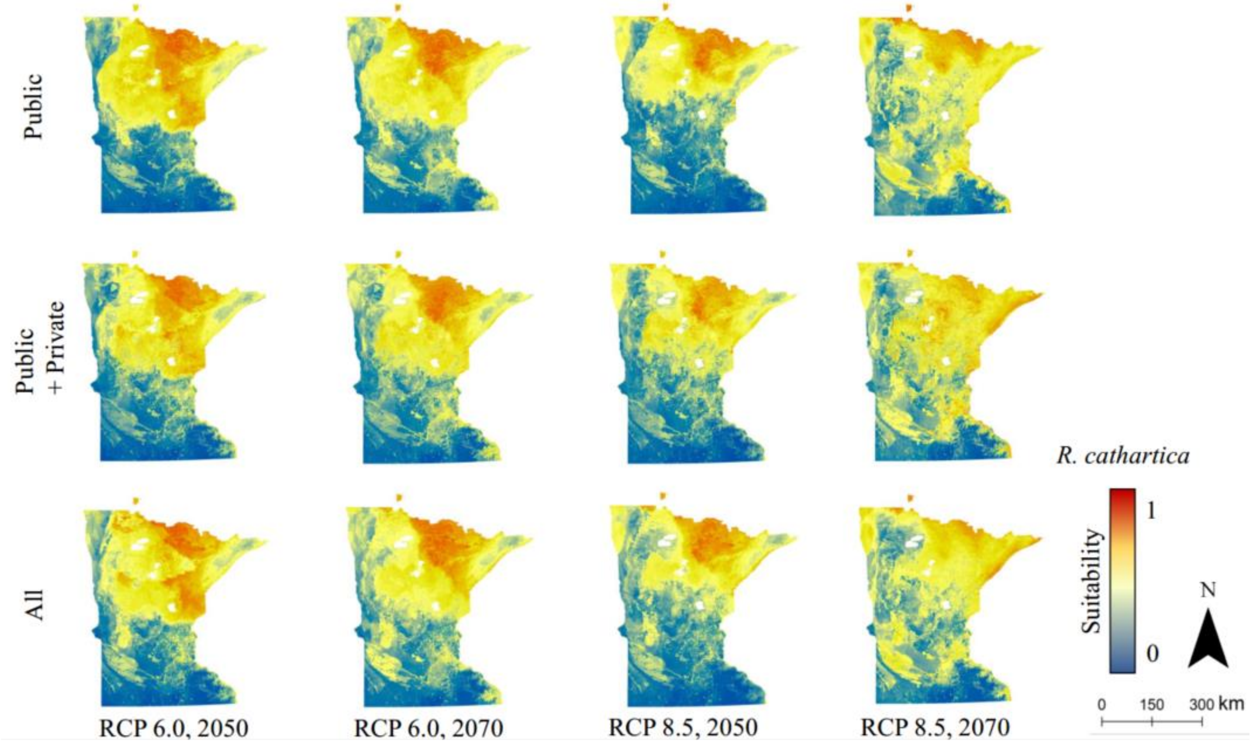

Figure 7. *R. cathartica* distribution model output across data source sets, for future climate conditions (RCP 6.0 and 8.5, 2050s and 2070s) under the CCSM climate model.

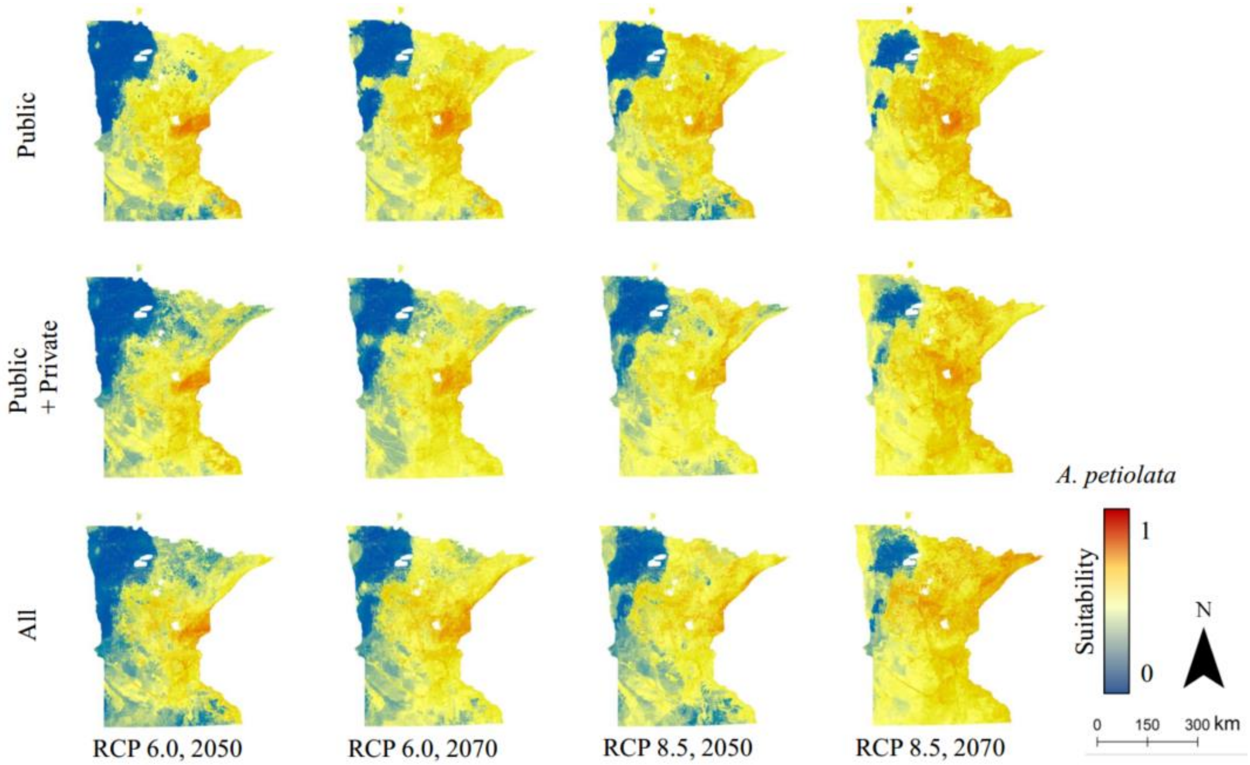

Figure 8. *A. petiolata* distribution model output across data source sets, for future climate conditions (RCP 6.0 and 8.5, 2050s and 2070s) under the CCSM climate model.

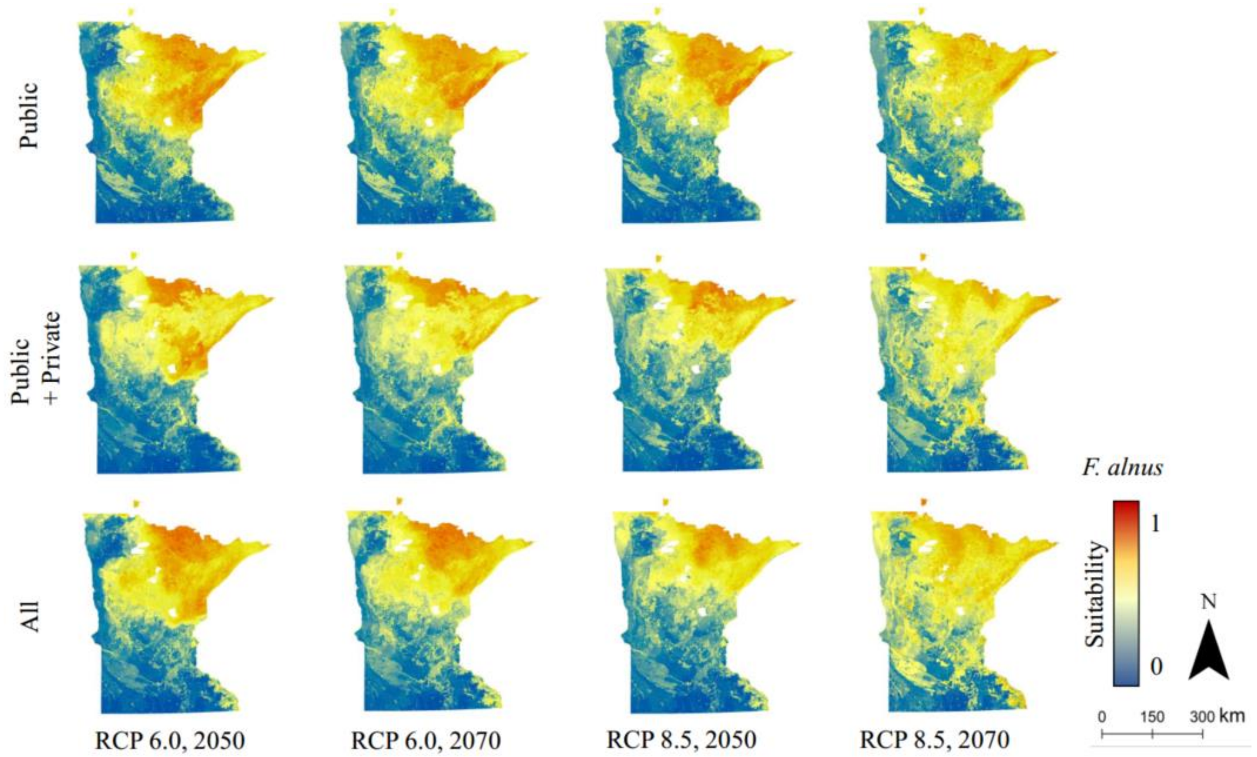

Figure 9. *F. alnus* distribution model output across data source sets, for future climate conditions (RCP 6.0 and 8.5, 2050s and 2070s) under the CCSM climate model.

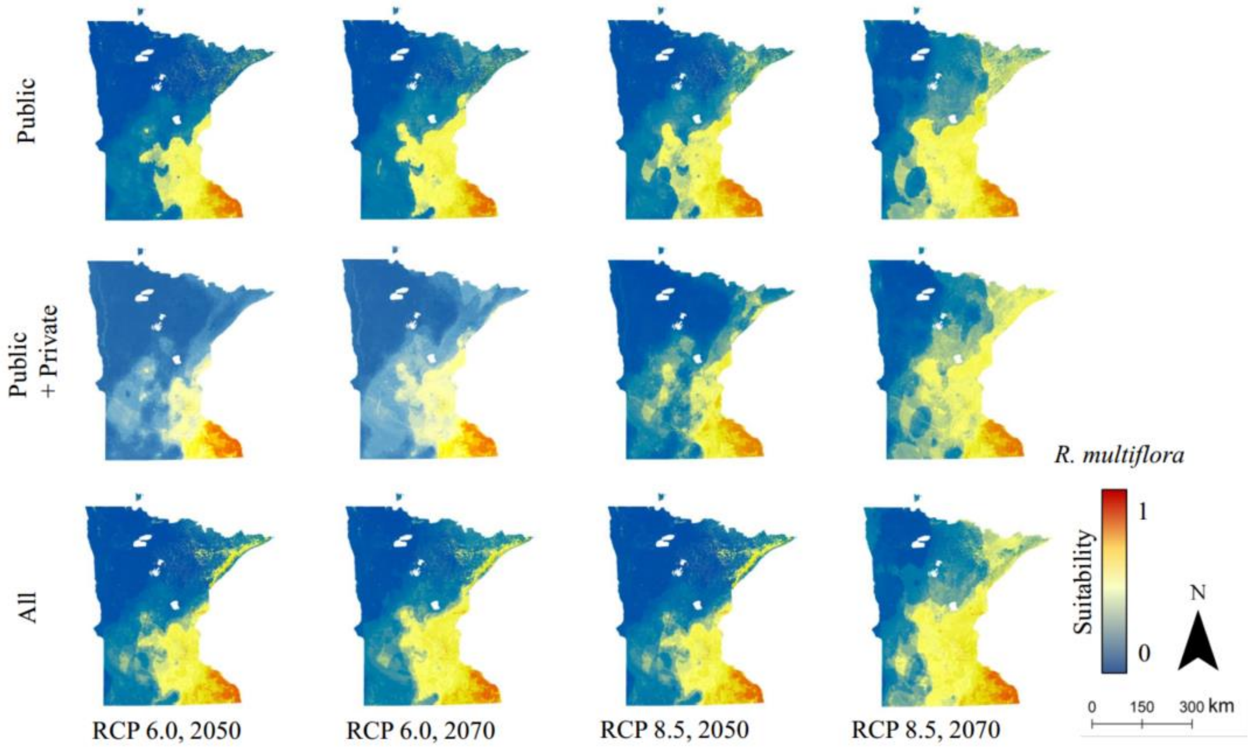

Figure 10. *R. multiflora* distribution model output across data source sets, for future climate conditions (RCP 6.0 and 8.5, 2050s and 2070s) under the CCSM climate model.

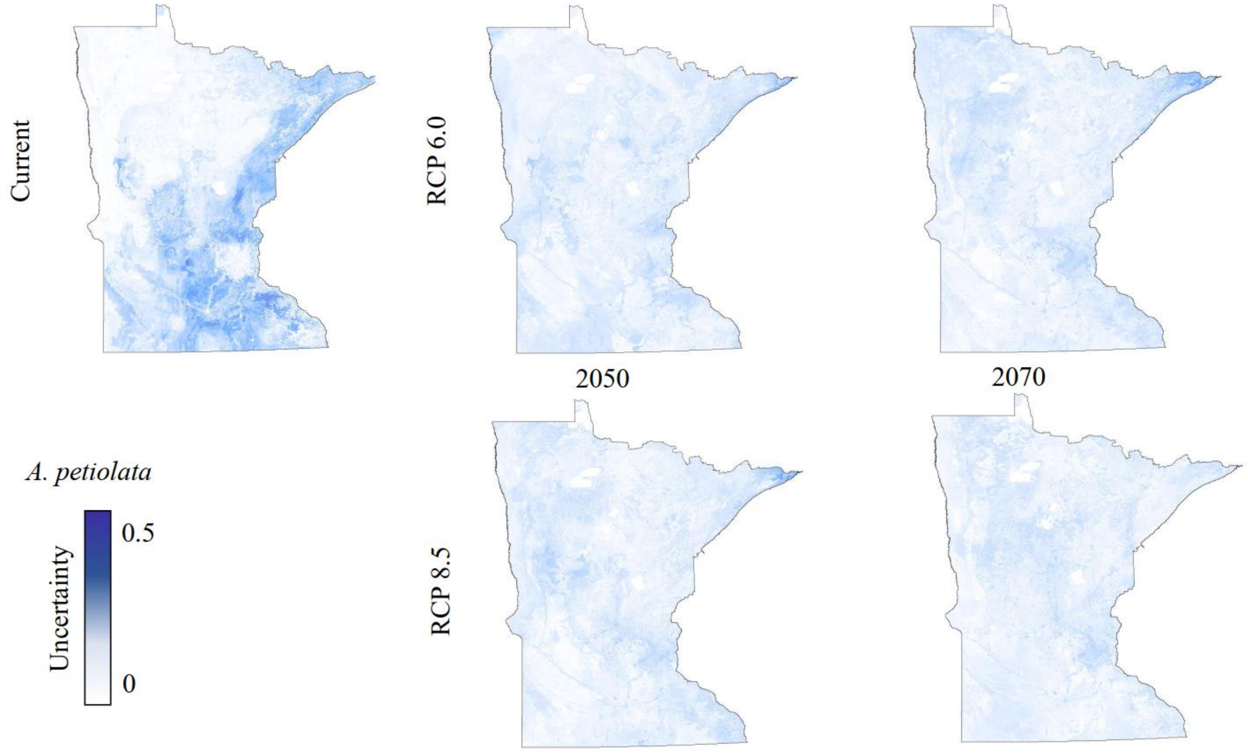

Figure 11. Distribution model uncertainty attributable to data set source, *A. petiolata.* Uncertainty is quantified here as the standard deviation between rasters of different data set source for the HadGEM climate scenarios.

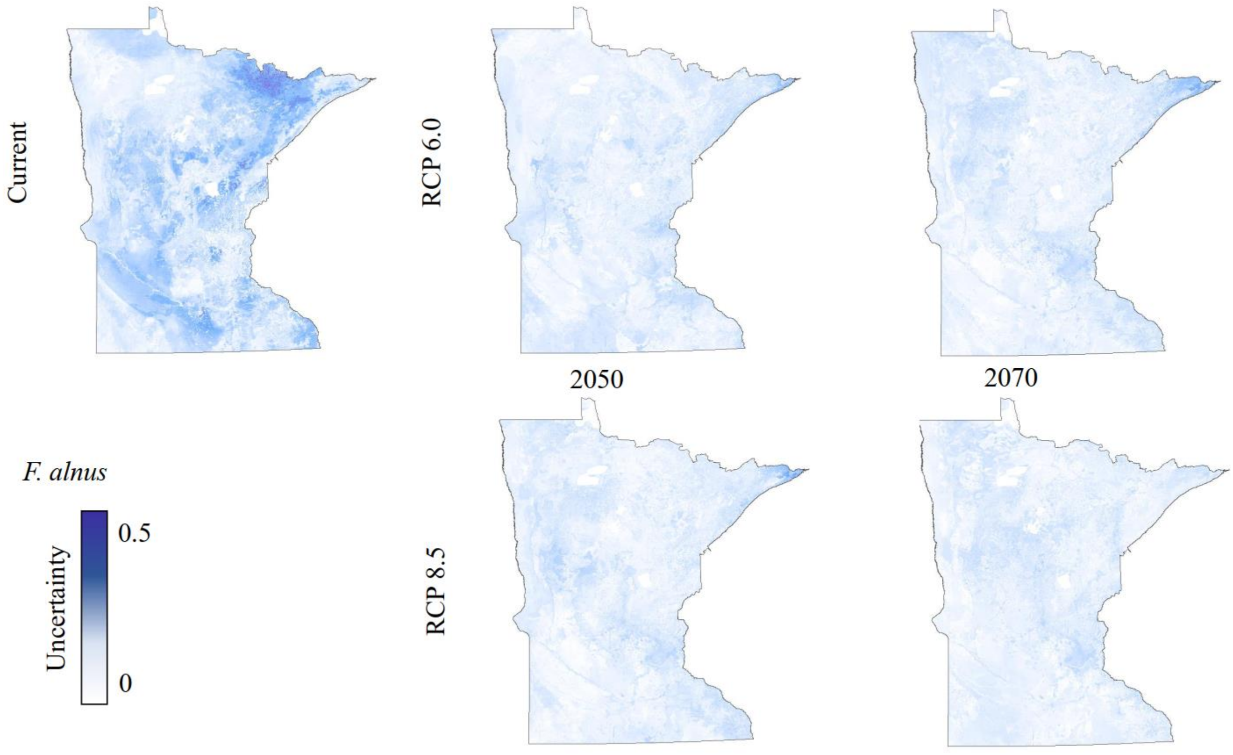

Figure 12. Distribution model uncertainty attributable to data set source, *F. alnus*. Uncertainty is quantified here as the standard deviation between rasters of different data set source for the HadGEM climate scenarios.

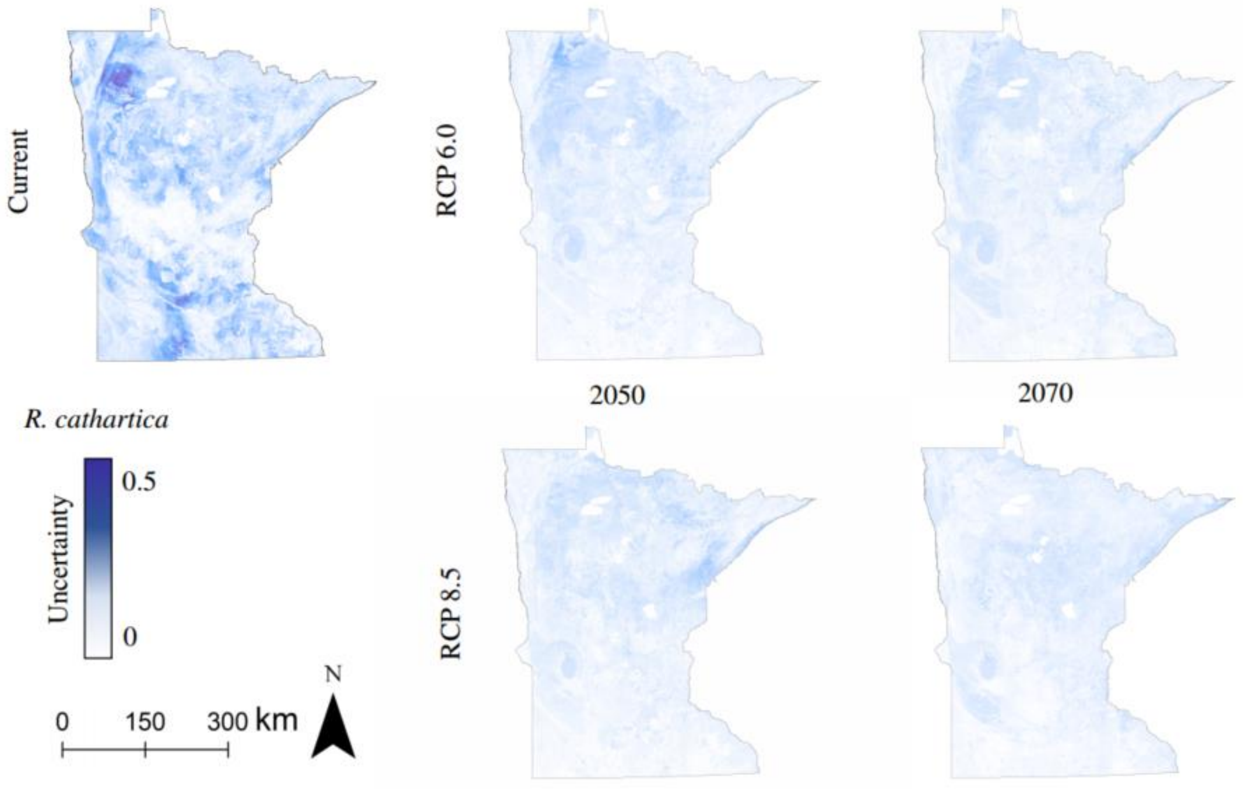

Figure 13. Distribution model uncertainty attributable to data set source, *R. cathartica*. Uncertainty is quantified here as the standard deviation between rasters of different data set source for the CCSM4 climate scenarios.

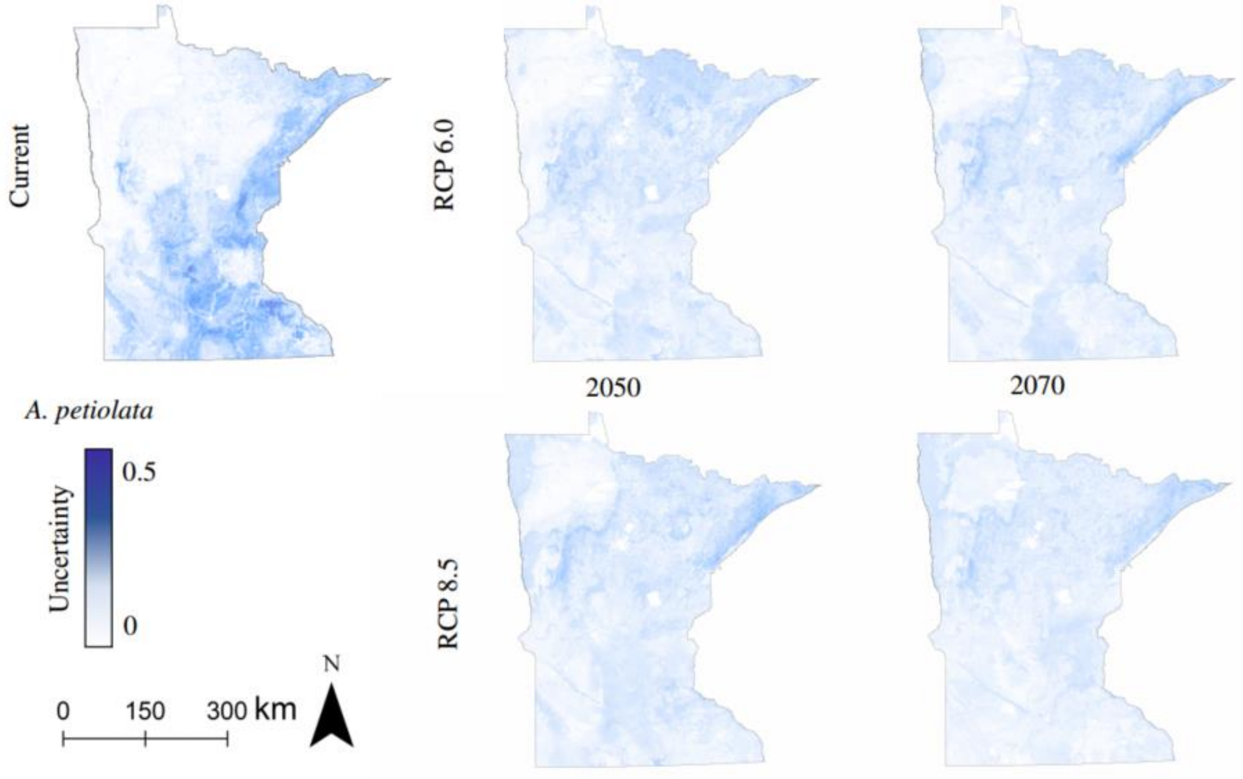

Figure 14. Distribution model uncertainty attributable to data set source, *A. petiolata*. Uncertainty is quantified here as the standard deviation between rasters of different data set source for the CCSM4 climate scenarios.

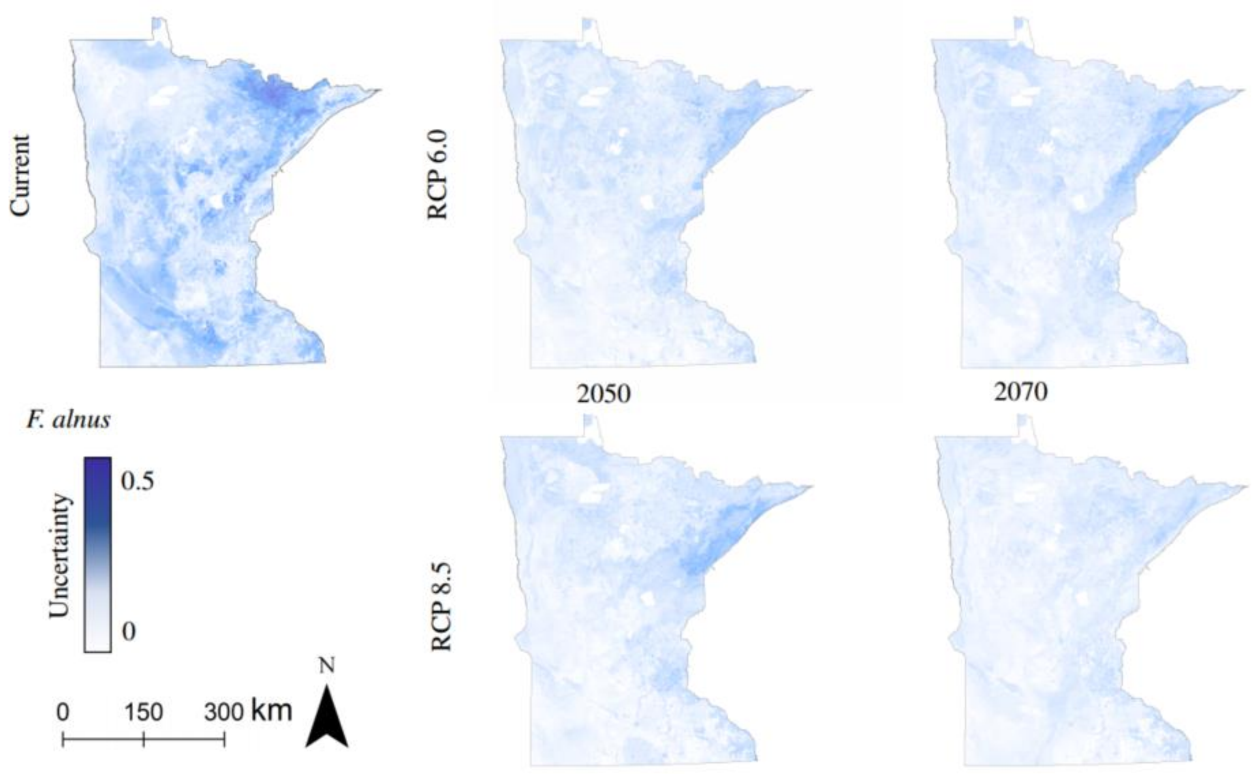

Figure 15. Distribution model uncertainty attributable to data set source, *F. alnus*. Uncertainty is quantified here as the standard deviation between rasters of different data set source for the CCSM4 climate scenarios.

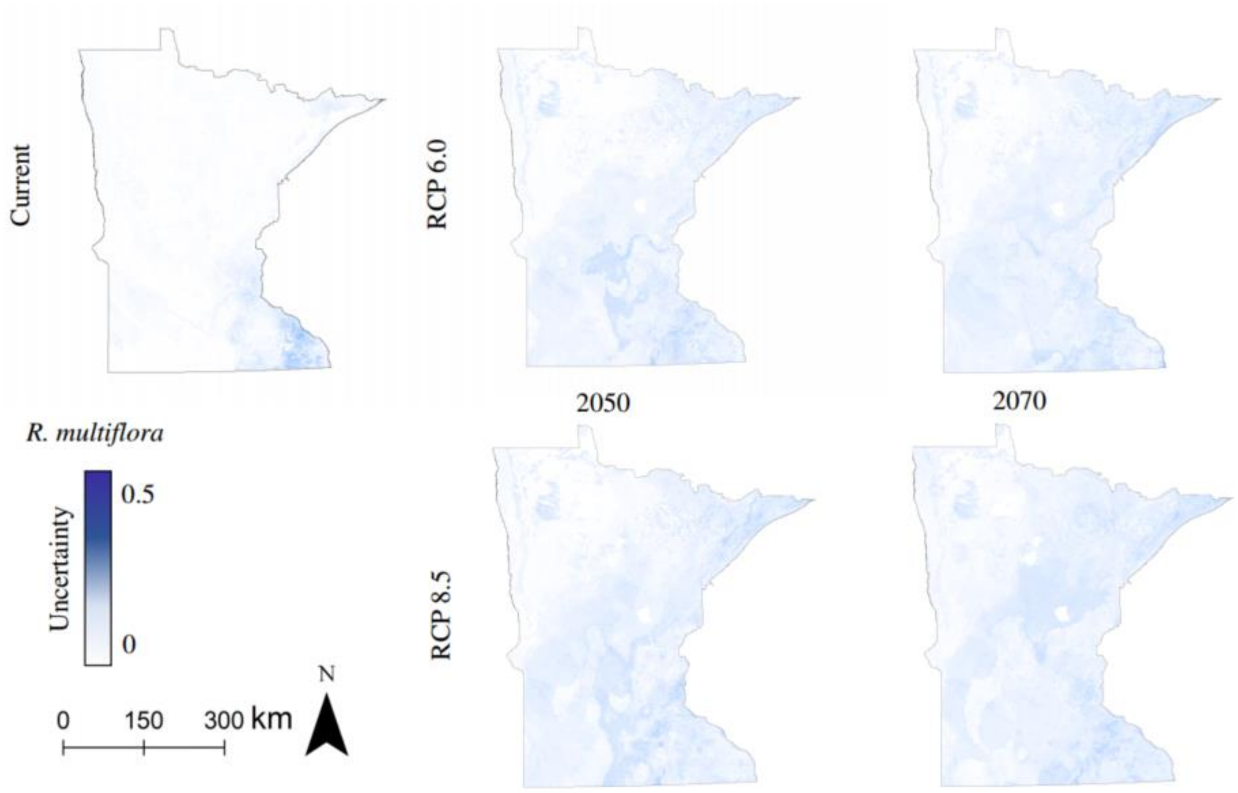

Figure 16. Distribution model uncertainty attributable to data set source, *R. multiflora.* Uncertainty is quantified here as the standard deviation between rasters of different data set source for the CCSM4 climate scenarios.

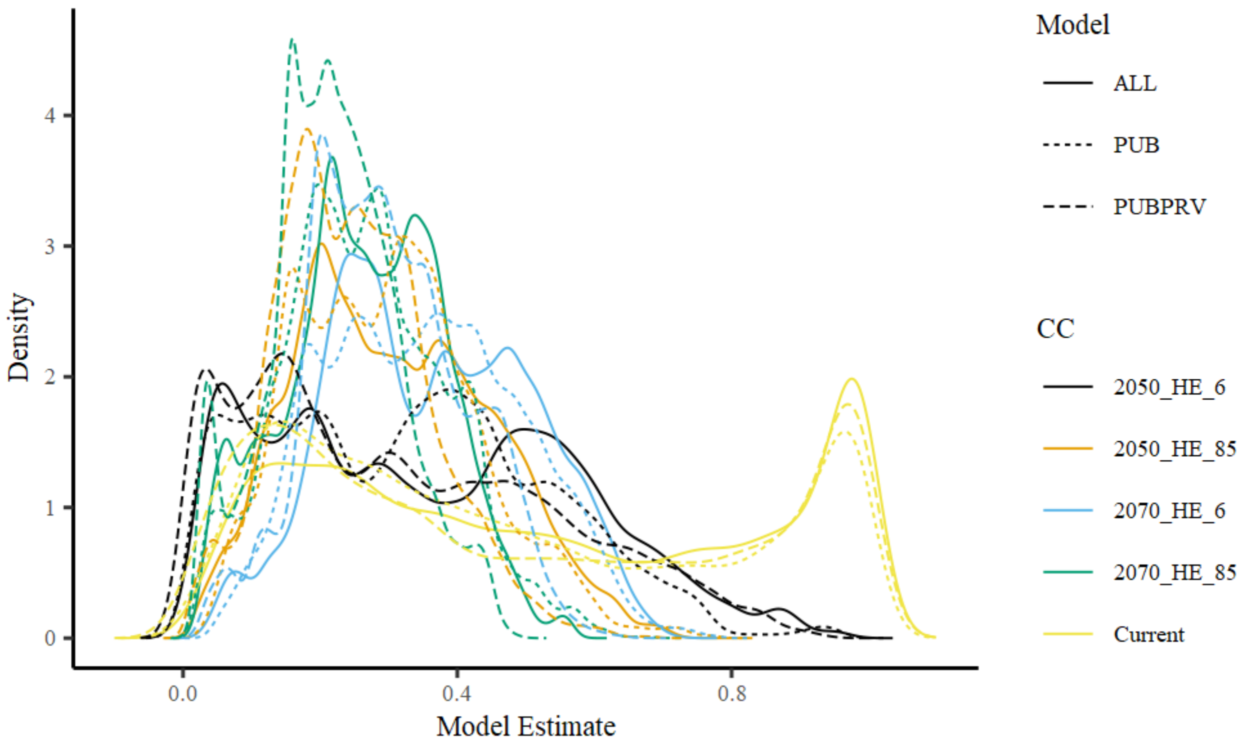

Figure 17. Density plot of model estimates for *R. cathartica* under the HadGEM climate model scenarios. Colors correspond to climate scenario and line type corresponds to data source model.

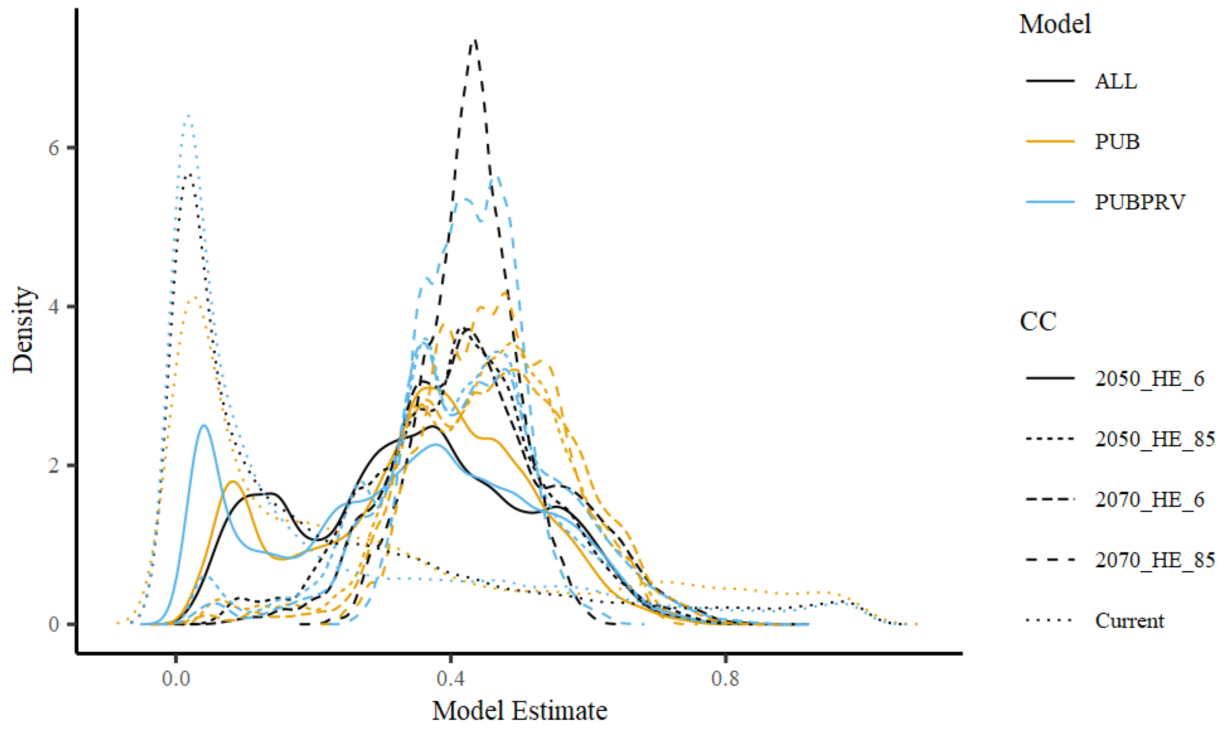

Figure 18. Density plot of model estimates for *A. petiolata* under the HadGEM climate model scenarios. Colors correspond to climate scenario and line type corresponds to data source model.

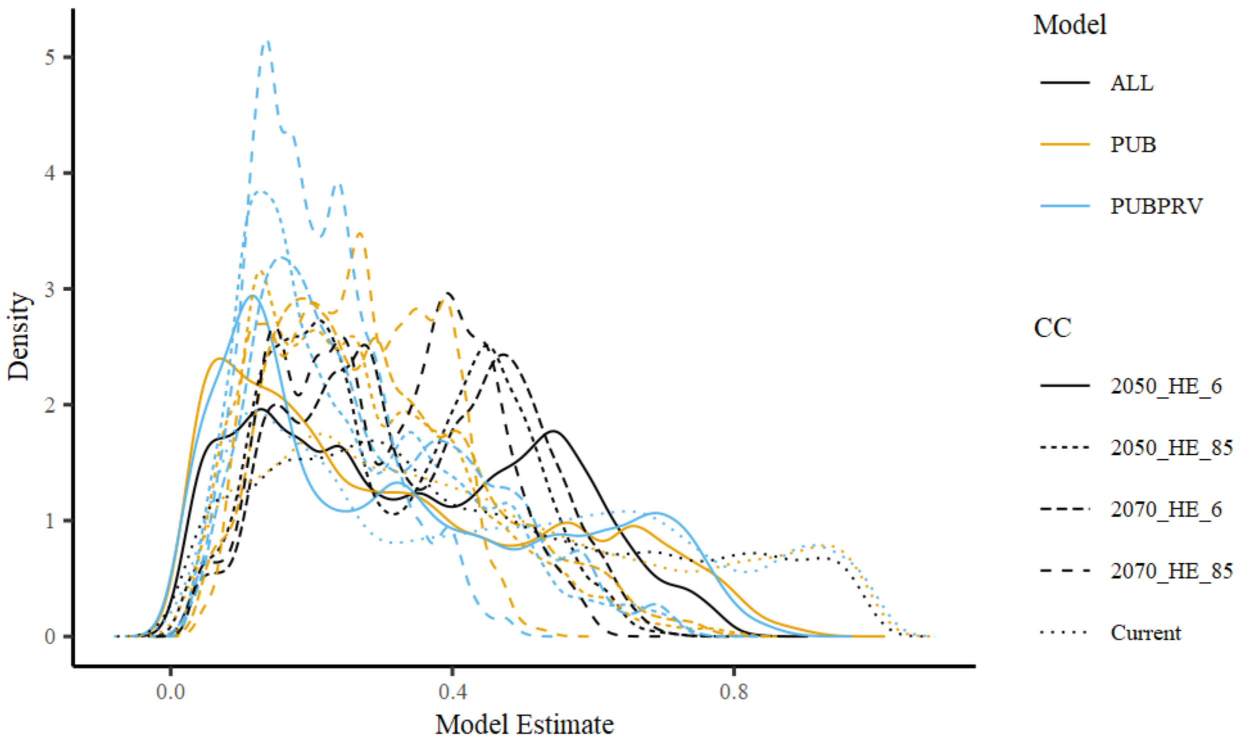

Figure 19. Density plot of model estimates for *F. alnus* under the HadGEM climate model scenarios. Colors correspond to climate scenario and line type corresponds to data source model.

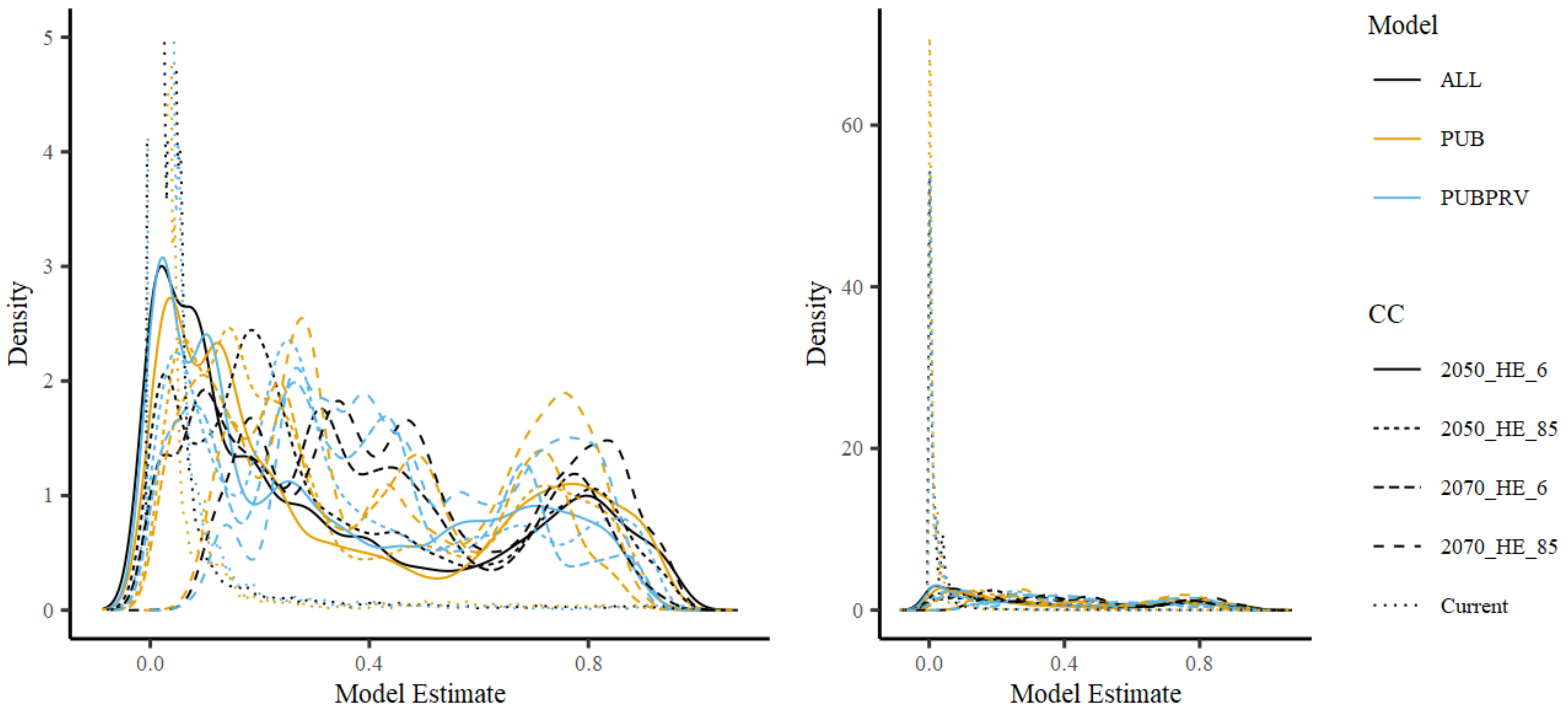

Figure 20. Density plot of model estimates for *R. multiflora* under the HadGEM climate model scenarios. Colors correspond to climate scenario and line type corresponds to data source model. Left-hand graph illustrates the truncated densities below 5.0 to highlight differences between climate change scenarios; right-hand graph illustrates the untruncated densities, highlighting the high density of low estimates for the current-day models.

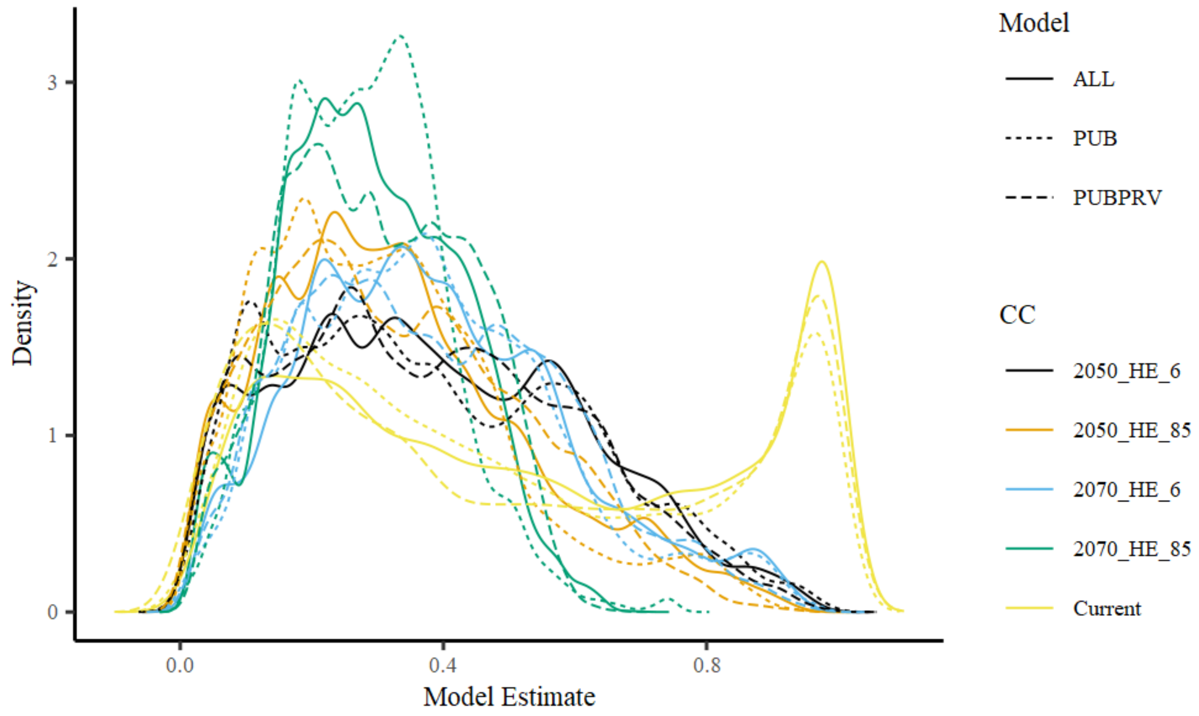

Figure 21. Density plot of model estimates for *R. cathartica* under the CCSM4 climate model scenarios. Colors correspond to climate scenario and line type corresponds to data source model.

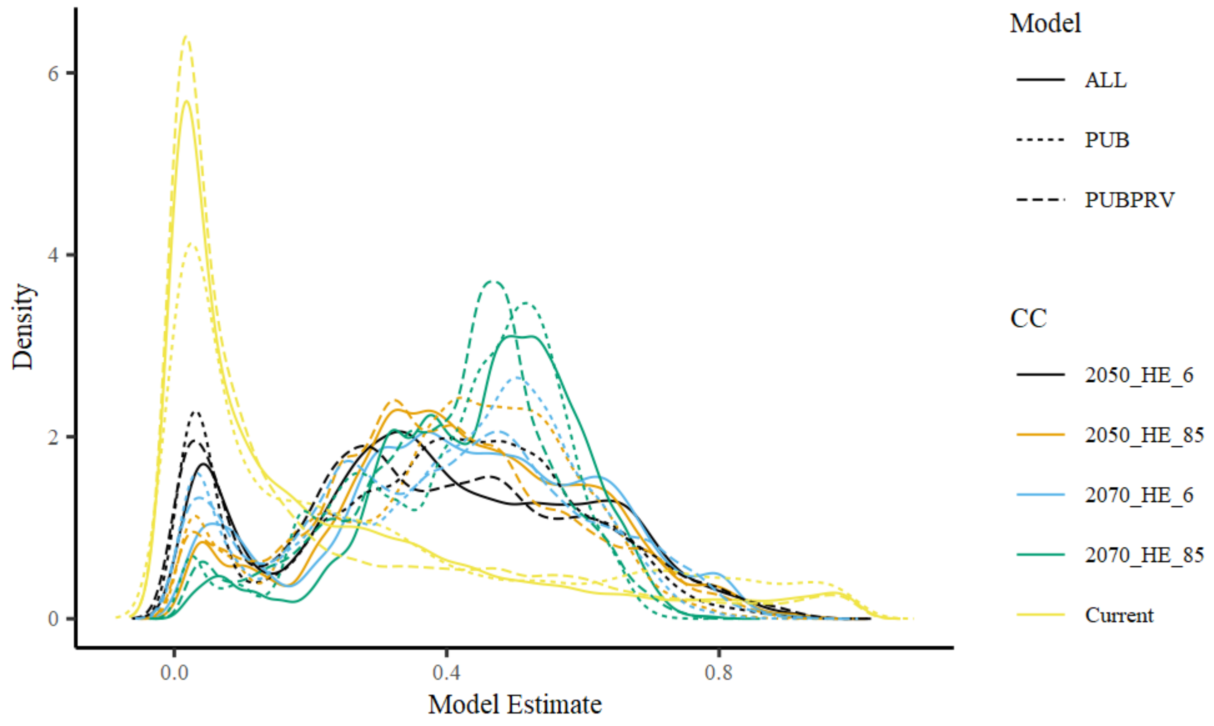

Figure 22. Density plot of model estimates for *A. petiolata* under the CCSM4 climate model scenarios. Colors correspond to climate scenario and line type corresponds to data source model.

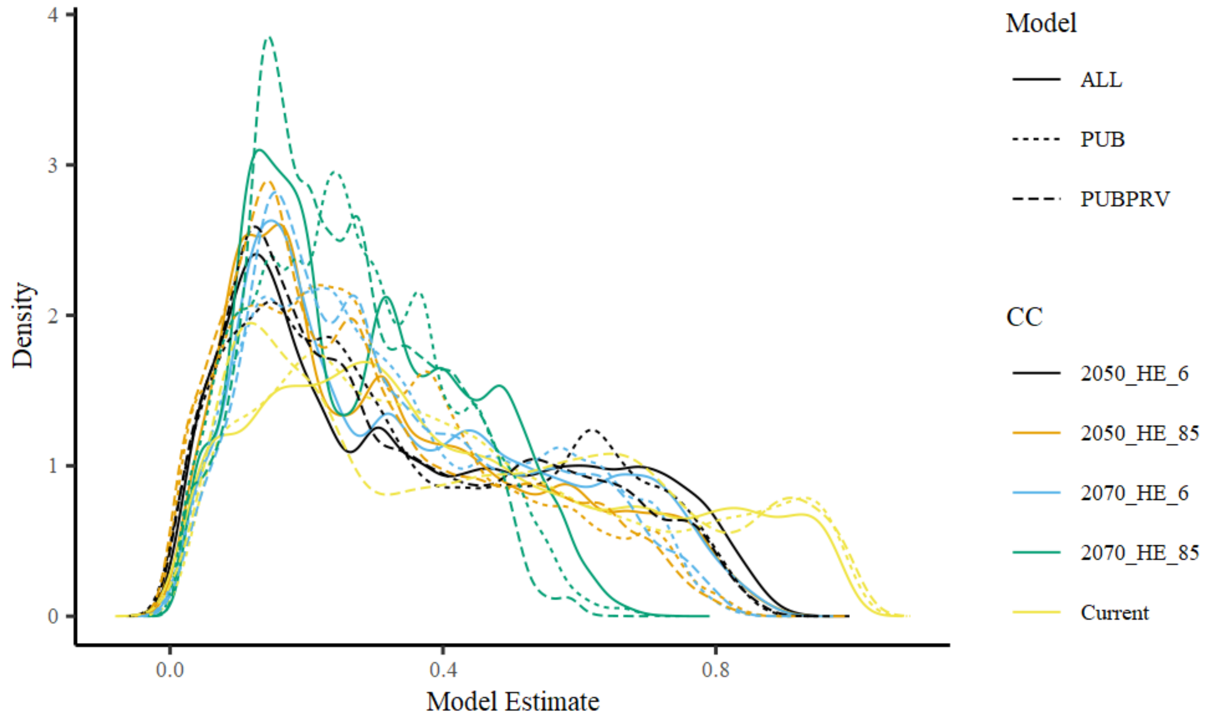

Figure 23. Density plot of model estimates for *F. alnus* under the CCSM4 climate model scenarios. Colors correspond to climate scenario and line type corresponds to data source model.

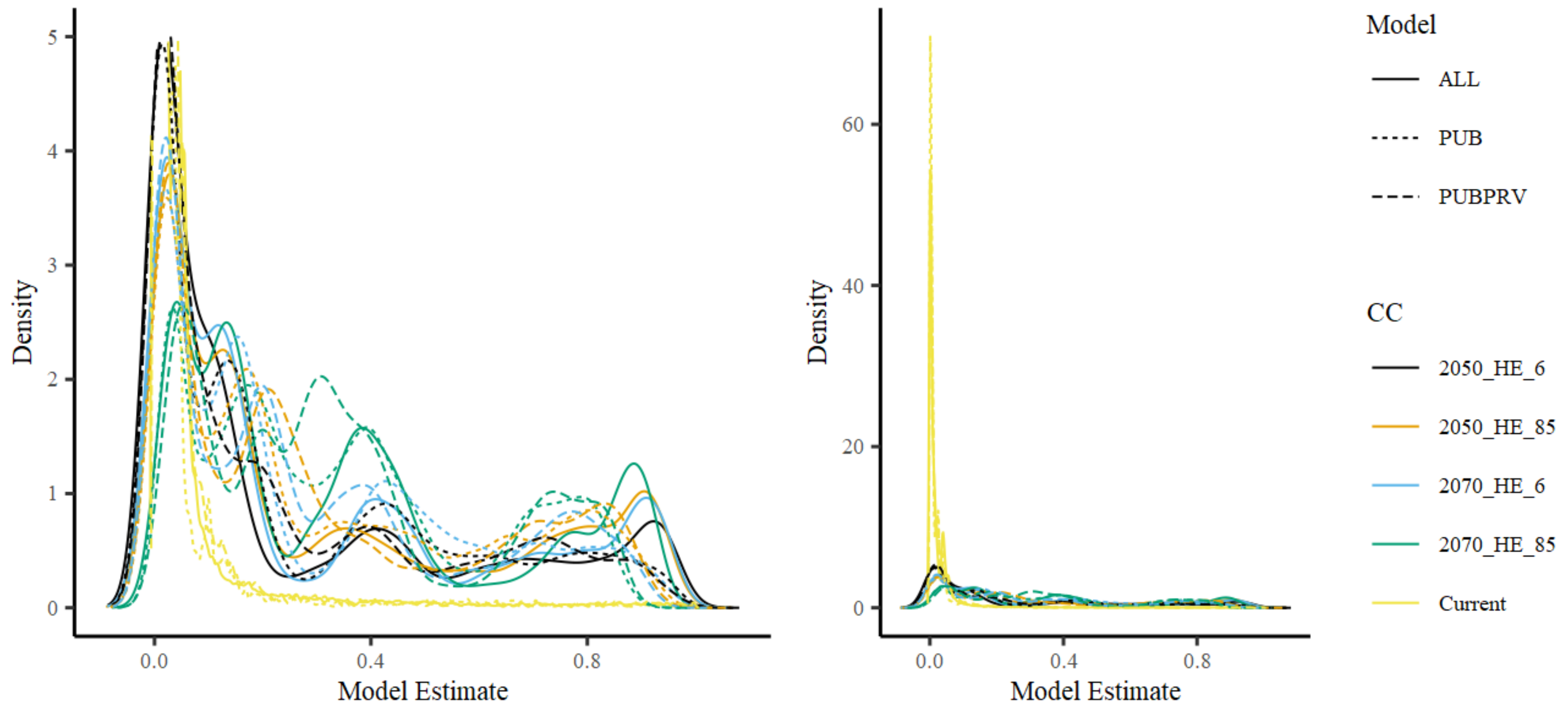

Figure 24. Density plot of model estimates for *R. multiflora* under the CCSM4 climate model scenarios. Colors correspond to climate scenario and line type corresponds to data source model. Left-hand graph illustrates the truncated densities below 5.0 to highlight differences between climate change scenarios; right-hand graph illustrates the untruncated densities, highlighting the high density of low estimates for the current-day models.
